# New Chlorinated 2,5-Diketopiperazines from Marine-Derived Bacteria Isolated from Sediments of the Eastern Mediterranean Sea

**DOI:** 10.3390/molecules25071509

**Published:** 2020-03-26

**Authors:** Maria Harizani, Eleni Katsini, Panagiota Georgantea, Vassilios Roussis, Efstathia Ioannou

**Affiliations:** Section of Pharmacognosy and Chemistry of Natural Products, Department of Pharmacy, School of Health Sciences, National and Kapodistrian University of Athens, Panepistimiopolis Zografou, Athens 15771, Greece; mariachariz@pharm.uoa.gr (M.H.); ekatsini@pharm.uoa.gr (E.K.); ggeorgantea@yahoo.gr (P.G.); roussis@pharm.uoa.gr (V.R.)

**Keywords:** 2,5-diketopiperazine, marine bacteria, sediment, natural products, structure elucidation, antifungal activity evaluation

## Abstract

From the organic extracts of five bacterial strains isolated from marine sediments collected in the East Mediterranean Sea, three new (**15**, **16**, **31**) and twenty-nine previously reported (**1**–**14**, **17**–**30**, **32**) metabolites bearing the 2,5-diketopiperazine skeleton were isolated. The structures of the chlorinated compounds **15**, **16**, and **31** were elucidated by extensive analysis of their spectroscopic data (NMR, MS, UV, IR). Compounds **15** and **16** were evaluated for their antifungal activity against *Candida albicans* and *Aspergillus niger* but were proven inactive. The relevant literature is supplemented with complete NMR assignments and revisions for the 29 previously reported compounds.

## 1. Introduction

2,5-Diketopiperazines (DKPs), also termed as cyclodipeptides, 2,5-dioxopiperazines, or dipeptide anhydrides, are the smallest possible cyclic peptides and, therefore, are among the most common peptide derivatives found in nature. They derive from the condensation of two α-amino acids forming a bis-lactam. Although they are relatively simple and low molecular weight compounds, they can be highly substituted, resulting in complex structures [1,2]. DKPs have been reported, so far, from a variety of sources, including microorganisms (bacteria and fungi), as well as higher organisms (algae, lichens, plants, marine sponges, gorgonians, tunicates, and mammals) [3,4]. They are also found in food and beverages, lending them a bitter taste [5]. The origin of DKPs has been questioned and it has been proposed that they might even be chemical degradation products. However, sterile media do not contain DKPs [6,7,8,9] and specific bacterial genes encode their biosynthesis [10,11,12].

DKPs have been neglected for many years, but they have recently attracted attention due to their chemical diversity and remarkable bioactivity. They exhibit a wide range of biological activities, such as cytotoxic, antibacterial, antifungal, antiparasitic, insecticidal, antiviral, antiprion, antifouling, antioxidant, anti-inflammatory, antihyperglycemic, and neuroprotective; thus, making them promising drug candidates. Moreover, they are involved in quorum sensing and ion-transport, and exhibit high binding affinity to a large number of receptors [4,13,14,15,16,17].

The DKP chiral scaffold is attractive for drug design due to its simplicity, high stability (resistance to proteolysis), conformational rigidity, and remarkable structural diversity [18]. DKPs are readily accessible by chemical synthesis, constituting an excellent model for theoretical studies and an important pharmacophore in medicinal chemistry [4,13,19]. Moreover, they are employed as starting materials for the synthesis of many natural products, such as alkaloids [18].

Three drugs based on this scaffold have recently entered the market, namely tadalafil, as a phosphodiesterase-5 inhibitor for the treatment of erectile dysfunction [20], retosiban, as an oxytocin antagonist for the treatment of preterm labor [21], and epelsiban, as an oxytocin antagonist for the treatment of premature ejaculation in men [22]. Additionally, it has been shown that their presence in culture broths fermented with lactic acid bacteria (LAB) can greatly contribute to an environmentally friendly, safe, and ecological approach for food and feed preservation [23].

**Figure 1:**
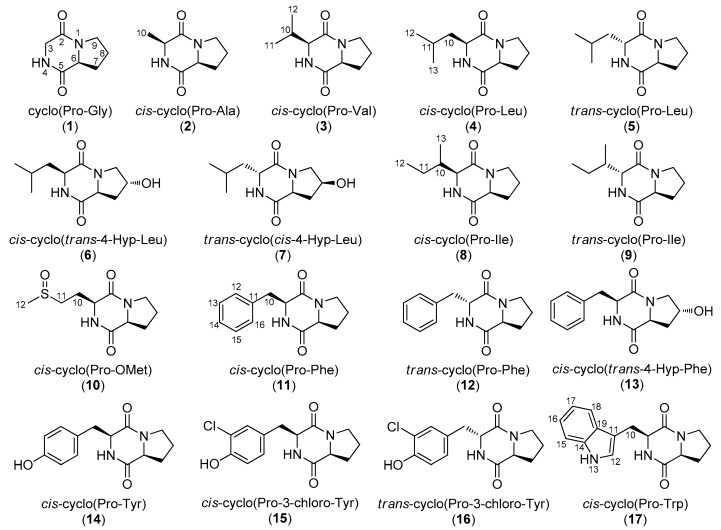
Chemical structures of compounds **1**–**17** isolated from various marine-derived bacterial strains.

**Figure 2:**
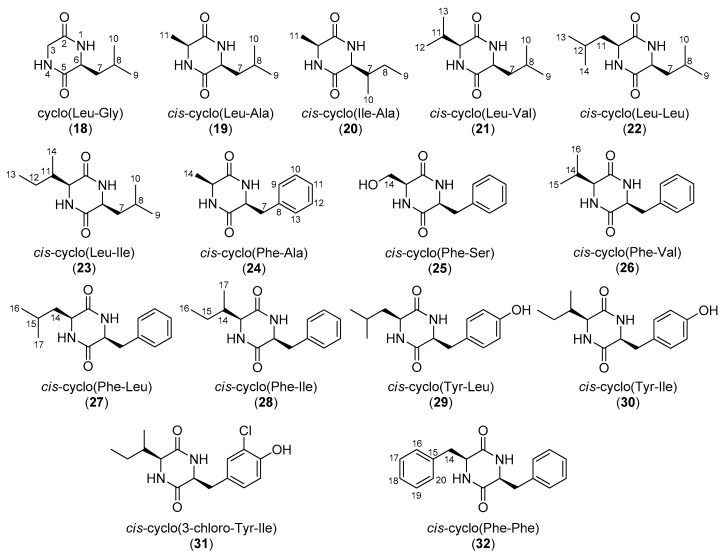
Chemical structures of compounds **18**–**32** isolated from various marine-derived bacterial strains.

In the framework of our investigations towards the isolation of new bioactive secondary metabolites from marine microorganisms, a large number of bacterial strains have been isolated from marine sediments collected from the East Mediterranean basin, a relatively unexplored marine ecosystem regarding the chemistry of its microbiota. The preliminary screening of the chemical profiles of extracts obtained from small-scale liquid cultures of a large number of marine-derived bacterial strains from our microbank with LC-DAD-MS and NMR led to the selection of five strains for further chemical investigation. Extraction of large-scale cultures of the selected bacterial strains and fractionation of the obtained organic extracts allowed for the isolation of 32 DKPs (Figure 1 and Figure 2), among which three chlorinated analogues (**15**, **16**, and **31**) were identified as new natural products. Herein, we report the isolation and structure elucidation of metabolites **15**, **16**, and **31** and the evaluation of the antifungal activities of **15** and **16**. Additionally, since several inconsistencies in the published NMR data of DKP structures are frequently observed, in conjunction with the fact that NMR data is incompletely reported for several of these, leading to confusion, complete assignment of the ^1^H and ^13^C NMR chemical shifts of the known metabolites **1**–**14**, **17**–**30**, and **32** is also presented.

## 2. Results and Discussion

The organic extracts of five marine-derived bacterial strains, specifically *Bacillus endophyticus* BI0327, *Streptomyces albidoflavus* BI0383, *Nocardiopsis aegyptia* BI0618, *Streptomyces smyrnaeus* BI0918, and *Bacillus subtilis* BI0980, isolated from marine sediments collected in the Aegean and Ionian seas, were subjected to repetitive chromatographic fractionations to yield three new natural products, namely *cis*-cyclo(Pro-3-chloro-Tyr) (**15**), *trans*-cyclo(Pro-3-chloro-Tyr) (**16**), and *cis*-cyclo(3-chloro-Tyr-Ile) (**31**), and 29 previously reported metabolites, which were identified as cyclo(Pro-Gly) (**1**) [24,25], *cis*-cyclo(Pro-Ala) (**2**) [26,27], *cis*-cyclo(Pro-Val) (**3**) [28,29,30], *cis*-cyclo(Pro-Leu) (**4**) [25,29,31,32], *trans*-cyclo(Pro-Leu) (**5**) [31,33], *cis*-cyclo(*trans*-4-Hyp-Leu) (**6**) [34], *trans*-cyclo(*cis*-4-Hyp-Leu) (**7**) [35], *cis*-cyclo(Pro-Ile) (**8**) [28,31,36], *trans*-cyclo(Pro-Ile) (**9**) [36,37], *cis*-cyclo(Pro-OMet) (**10**) [38], *cis*-cyclo(Pro-Phe) (**11**) [28,30,31,39,40], *trans*-cyclo(Pro-Phe) (**12**) [33,36,40], *cis*-cyclo(*trans*-4-Hyp-Phe) (**13**) [36,41], *cis*-cyclo(Pro-Tyr) (**14**) [42,43], *cis*-cyclo(Pro-Trp) (**17**) [44,45], cyclo(Leu-Gly) (**18**) [46,47,48], *cis*-cyclo(Leu-Ala) (**19**) [49,50], *cis*-cyclo(Ile-Ala) (**20**) [49], *cis*-cyclo(Leu-Val) (**21**) [37], *cis*-cyclo(Leu-Leu) (**22**) [37,51,52], *cis*-cyclo(Leu-Ile) (**23**) [17], *cis*-cyclo(Phe-Ala) (**24**) [49], *cis*-cyclo(Phe-Ser) (**25**) [53], *cis*-cyclo(Phe-Val) (**26**) [17], *cis*-cyclo(Phe-Leu) (**27**) [17,54], *cis*-cyclo(Phe-Ile) (**28**) [17], *cis*-cyclo(Tyr-Leu) (**29**) [54], *cis*-cyclo(Tyr-Ile) (**30**) [17], and *cis*-cyclo(Phe-Phe) (**32**) [55] by comparison of their spectroscopic and physical characteristics with those reported in the literature.

**Figure 3:**
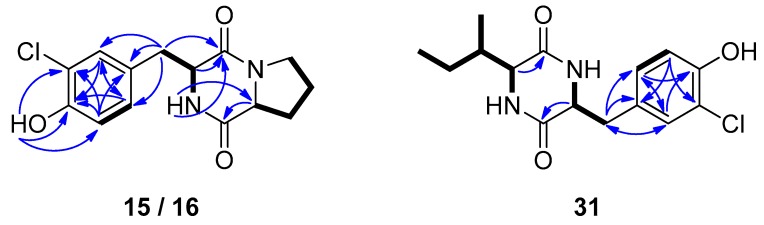
COSY (bold bonds) and important HMBC (arrows) correlations observed for compounds **15**/**16** and **31**.

Compound **15**, obtained as white solid, displayed the molecular formula C_14_H_15_N_2_O_3_Cl, as deduced from high-resolution electrospray ionization mass spectrometry (HRESIMS) measurements where two isotopic sodium adduct ion peaks were observed at *m*/*z* 317.0661 and 319.0629 with a ratio of 3:1, characteristic for the presence of one chlorine atom in the molecule. The HSQC and HMBC experiments revealed 14 carbon signals, which corresponded to five non-protonated carbon atoms, among which two carbonyls resonating at *δ*_C_ 164.7 and 169.1, five methines, and four methylenes. The ^1^H and ^13^C NMR spectra (Table 1 and Table 2 and Figures S1–S6) included signals at *δ*_H_ 6.98 (1H, d, 8.2 Hz), 7.03 (1H, dd, 8.2, 2.0 Hz), and 7.18 (1H, d, 2.0 Hz) indicative of a 1,2,4-trisubstituted aromatic ring, whereas signals for two deshielded methines at *δ*_H/C_ 4.07/59.0 and 4.21/55.8 and one exchangeable proton signal at *δ*_H_ 5.55, pointing to a DKP skeleton, were observed. The COSY correlations of H-6/H_2_-7, H_2_-7/H_2_-8, and H_2_-8/H_2_-9 supported the presence of a proline moiety, while further interpretation of the HMBC data unambiguously connected the spin systems (Figure 3) and verified the planar structure of **15**. The NOE correlation of H-3 and H-6 determined their *cis* orientation and assigned the relative configuration of **15** that was identified as *cis*-cyclo(Pro-3-chloro-Tyr). Compound **15**, described here for the first time as a natural product, has been previously reported as a synthetic derivative [43].

Compound **16**, which also displayed two sodium adduct ion peaks at *m*/*z* 317.0657 and 319.0627 with a ratio of 3:1 (HRESIMS), was isolated as white solid. The spectroscopic characteristics of **16** (Table 1 and Table 2 and Figures S7–S12) were rather similar to those of **15**. Specifically, the NMR spectra of **16** revealed the same structural characteristics of a DKP moiety, including a proline amino acid and a 1,2,4-trisubstituted aromatic ring. The most prominent difference was that H-3 (4.13 ppm) and H-6 (3.22 ppm) resonated in higher fields, which, in combination with the absence of an NOE correlation between them, indicated that compound **16** was the *trans* isomer of **15**. The COSY cross-peaks and the HMBC correlations observed for **16** (Figure 3), in accordance to those observed for compound **15**, were in agreement with the proposed structure of *trans*-cyclo(Pro-3-chloro-Tyr).

Compound **31**, was obtained in trace amounts as a 1:1 mixture with compound **30**. The gas chromatography – electron ionization mass spectrometry (GC-EIMS) chromatogram included two peaks, the first displaying a molecular ion peak [M]^+^ at *m*/*z* 276 and a fragmentation pattern identical to that of *cis*-cyclo(Tyr-Ile) (**30**), whereas the second displayed molecular ion peaks [M]^+^ at *m*/*z* 310 and 312 with an isotopic ratio of 3:1, suggesting that **31** was a monochlorinated compound. Comparison of the ^1^H NMR data of the mixture with that of *cis*-cyclo(Tyr-Ile) (**30**) in pure form revealed the structural similarity of metabolites **31** and **30**, with the main difference observed in the aromatic ring (Table 1 and Table 2 and Figures S13–S17). Indeed, in the aromatic region of the ^1^H NMR spectrum, the signals at *δ*_H_ 6.97 (1H, d, 8.3 Hz), 7.02 (1H, br d, 8.3 Hz), and 7.17 (1H, br s), indicative of a 1,2,4-trisubstituted aromatic ring, were assigned to compound **31**, whereas two signals at *δ*_H_ 6.79 (d, 8.0 Hz) and 7.07 (d, 8.0 Hz), integrating for two protons each, were assigned to the protons of the *p*-substituted aromatic ring of compound **30**. Analysis of the 2D NMR spectra (Figure 3) confirmed the residue of isoleucine and the proposed planar structure of **31**, whereas comparison of the chemical shifts of H-3 (*δ* 3.90) and H-6 (*δ* 4.19) to those of compound **30** indicated their *cis* orientation. Thus, compound **31** was identified as *cis*-cyclo(3-chloro-Tyr-Ile).

Since several inconsistencies are observed for the published NMR data of frequently isolated DKPs, in conjunction to the fact that NMR data are incompletely reported for a number of them, the ^1^H and ^13^C NMR data for the known compounds **1**–**14**, **17**–**30**, and **32** are presented in Table 1 and Table 2, complementing and revising the relevant literature data. Through careful analysis of the ^13^C NMR chemical shifts of the proline-containing *cis*/*trans* pairs **4**/**5**, **6**/**7**, **8**/**9**, **11**/**12**, and **15**/**16**, it can be observed that the chemical shifts of C-3 and C-10 are consistently deshielded by 3 and 3.5–4.5 ppm, respectively, in the *trans* DKP isomers.

Compounds **15** and **16** were evaluated for their antifungal activity against *Candida albicans* and *Aspergillus niger*. However, neither of the two metabolites exerted any significant effect on the growth of the two fungal strains.

## 3. Materials and Methods

### 3.1. General Experimental Procedures

Optical rotations were measured on a Krüss model P3000 polarimeter (A. KRÜSS Optronic GmbH, Hamburg, Germany) with a 0.5 dm cell. UV spectra were obtained on a Perkin Elmer Lambda 40 spectrophotometer (PerkinElmer Ltd., Buckinghamshire, UK). IR spectra were obtained on a Bruker Alpha II spectrometer (Bruker Optik GmbH, Ettlingen, Germany). 1D and 2D NMR spectra were recorded on Bruker DRX 400, Avance NEO 700 and Avance NEO 950 (Bruker BioSpin GmbH, Rheinstetten, Germany) and Varian 600 (Varian, Inc., Palo Alto, CA, USA), spectrometers, using standard Bruker or Varian pulse sequences at room temperature. Chemical shifts are given on a *δ* (ppm) scale using TMS as internal standard. High-resolution electrospray ionization (ESI) mass spectra were measured on a Thermo Scientific LTQ Orbitrap Velos mass spectrometer (Thermo Fisher Scientific, Bremen, Germany). Low-resolution electron ionization (EI) mass spectra were measured on a Hewlett-Packard 5973 mass spectrometer (Agilent Technologies, Santa Clara, CA, USA) or on a Thermo Electron Corporation DSQ mass spectrometer (Thermo Electron Corporation, Austin, TX, USA). Normal- and reversed-phase column chromatography separations were performed with Kieselgel Si 60 (Merck, Darmstadt, Germany) and Kieselgel RP-18 (Merck, Darmstadt, Germany), respectively. HPLC separations were conducted on (i) a Cecil 1100 Series liquid chromatography pump (Cecil Instruments Ltd., Cambridge, UK) equipped with a GBC LC-1240 refractive index detector (GBC Scientific Equipment, Braeside, VIC, Australia), (ii) a Pharmacia LKB 2248 liquid chromatography pump (Pharmacia LKB Biotechnology, Uppsala, Sweden) equipped with an RI-102 Shodex refractive index detector (ECOM spol. s r.o., Prague, Czech Republic), (iii) an Agilent 1100 liquid chromatography system equipped with refractive index detector (Agilent Technologies, Waldbronn, Germany), (iv) a Waters 600 liquid chromatography pump (Waters, Milford, MA, USA) with a Waters 410 refractive index detector (Waters, Milford, MA, USA), or (v) a Waters 515 liquid chromatography pump (Waters, Milford, MA, USA) equipped with a Shimadzu RID-20A refractive index detector (Shimadzu Europa GmbH, Duisburg, Germany), using the following columns: (i) Econosphere C_18_ 10u (250 × 10 mm, Grace, Columbia, MD, USA), (ii) Kromasil 100-7-C_18_ (250 × 10 mm, Akzonobel, Eka Chemicals AB, Separation Products, Bohus, Sweden), (iii) Luna C_18_ (2) 100A 10u (250 × 10 mm, Phenomenex, Torrance, CA, USA), (iv) Econosphere Silica 10u (250 × 10 mm, Grace, Columbia, MD, USA), (v) Kromasil 100-10-SIL (250 × 10 mm, Akzonobel, Eka Chemicals AB, Separation Products, Bohus, Sweden), or (vi) Supelcosil SPLC-Si 5 μm (250 × 10 mm, Supelco, Bellefonte, PA, USA). TLC was performed with Kieselgel 60 F_254_ aluminum-backed plates (Merck, Darmstadt, Germany) and spots were visualized after spraying with 15% (v/v) H_2_SO_4_ in MeOH reagent and heating at 100 °C for 1 min.

### 3.2. Biological Material

The bacterial strains were isolated from marine sediments collected from the East Mediterranean Sea and were identified based on comparison of their 16S ribosomal RNA (rRNA) sequences with data from the Genbank database of the National Center for Biotechnology Information (NCBI) using the Basic Local Alignment Search Tool (BLAST). Specifically, strain BI0327, identified as *Bacillus endophyticus* (GenBank accession number DQ485415), was isolated from a sediment collected east of Thiorichio in the island of Milos, at a depth of 4 m, in July 2012. Strain BI0383, identified as *Streptomyces albidoflavus* (GenBank accession number KJ573071), was isolated from a sediment collected east of Loutra in the island of Kythnos, at a depth of 150 m, in March 2013. Strain BI0618, identified as *Nocardiopsis aegyptia* (GenBank accession number NR_025589), was isolated from a sediment collected west of Agios Ioannis, in the island of Lemnos, at a depth of 6 m, in October 2013. Strain BI0918, identified as *Streptomyces smyrnaeus* (GenBank accession number NR_134201), was isolated from a sediment collected south of Vatsa, in the island of Kefalonia, at a depth of 75 m, in May 2014. Strain BI0980, identified as *Bacillus subtilis* (GenBank accession number JN560160), was isolated from a sediment collected in the waters between the islands of Kerkyra and Erikoussa, at a depth of 18 m, in August 2014. The strains have been deposited at the strain collection/microbank of the Section of Pharmacognosy and Chemistry of Natural Products, Department of Pharmacy, National and Kapodistrian University of Athens.

### 3.3. Fermentation, Extraction, and Isolation

The bacterial strain BI0327 was inoculated from a glycerol stock into a 1 L flask containing 500 mL of freshly prepared seawater-based (A1BFe+C) medium (10 g starch, 4 g yeast extract, 2 g peptone, 1 g CaCO_3_, 0.1 g KBr, and 0.04 g Fe_2_(SO4)_3_ 5H_2_O per liter of filtered seawater) [56]. After 7 days of incubation at 28 °C, while shaking at 120 rpm in an orbit shaker, the starter cultures were inoculated into 3 L flasks containing 1.5 L of the same seawater-based medium (4% *v*/*v* inoculum), to a total of 12 L of liquid medium, which were incubated at 28 °C for 14 days, while shaking at 120 rpm in an orbit shaker. Four days before the end of the fermentation period, Amberlite XAD-7HP resin (Sigma-Aldrich, St. Louis, MO, USA) (20 g/L) was added to each flask to adsorb extracellular metabolites. The broth was centrifuged and the pellet (resin and cell mass), was extracted twice for 24 h with Me_2_CO (8 L in total). Filtration of the extract and removal of the solvent under vacuum at 38 °C afforded a solid residue, which was partitioned between *n*-butanol and H_2_O. Evaporation of the solvent of the *n*-butanol soluble fraction in vacuo afforded a dark brown oily residue (2.2 g) that was subjected to vacuum column chromatography on silica gel, using cyclohexane, with increasing amounts of EtOAc, followed by EtOAc, with increasing amounts of MeOH as the mobile phase, to afford 8 fractions (327A–327H). Fraction 327G (50% MeOH in EtOAc, 1.1 g) was further fractionated by gravity column chromatography on silica gel, using EtOAc with increasing amounts of MeOH as the mobile phase, to yield 26 fractions (327G1-327G26). Fractions 327G10 to 327G15 (2% to 10% MeOH in EtOAc, 110.0 mg) were combined and purified by reversed-phase HPLC, using MeOH/H_2_O (70:30 and subsequently 50:50) and MeCN/H_2_O (30:70) as eluent, to afford **3** (3.9 mg), **4** (2.4 mg), **5** (1.7 mg), **6** (8.3 mg), **8** (6.6 mg), **11** (7.3 mg), **13** (6.1 mg), and **17** (1.2 mg). Fractions 327G16 (10% MeOH in EtOAc, 29.7 mg) and 327G17 (10% MeOH in EtOAc, 26.3 mg) were separately purified by reversed-phase HPLC, using MeOH/H_2_O (50:50) as eluent, and subsequently normal-phase HPLC, using cyclohexane/Me_2_CO (20:80) as eluent, to yield **7** (2.7 mg) and **13** (6.1 mg).

The bacterial strain BI0383 was inoculated from a glycerol stock into a 100 mL flask containing 50 mL of freshly prepared seawater-based (A1BFe+C) medium. After 4 days of incubation at 24 °C while shaking at 125 rpm in an orbit shaker, the starter culture was streaked onto 18 freshly prepared agar plates containing the same seawater-based medium. After 7 days, when sufficient growth of the bacterial strain was observed, mycelia were picked from the agar plates and were inoculated into 2 L flasks containing 1 L of the same seawater-based medium, to a total of 6 L of liquid medium, that were incubated at 27 °C for 7 days while shaking at 125 rpm in an orbit shaker. At the end of the fermentation period, Amberlite XAD-7HP resin (20 g/L) was added to each flask to adsorb extracellular metabolites. The culture and resin were shaken overnight at low speed. The broth was centrifuged and the pellet (resin and cell mass) was extracted twice for 24 h with Me_2_CO (4 L in total). Filtration of the extract and removal of the solvent under vacuum at 38 °C afforded a solid residue, which was partitioned between *n*-butanol and H_2_O. Evaporation of the solvent of the *n*-butanol soluble fraction in vacuo afforded a brown oily residue (2.22 g) that was subjected to vacuum column chromatography on silica gel, using cyclohexane with increasing amounts of EtOAc, followed by EtOAc with increasing amounts of MeOH as the mobile phase, to yield 12 fractions (383A–383L). Fraction 383K (10% MeOH in EtOAc, 54.0 mg) was submitted to normal-phase HPLC, using cyclohexane/Me_2_CO (55:45) as eluent, to afford **4** (6.0 mg), **3** (0.4 mg), and **8** (0.9 mg). The soluble in 80% MeOH in H_2_O part (111.0 mg) of fraction 383L (25% MeOH in EtOAc) was purified by reversed-phase HPLC, using MeCN/H_2_O (10:90) as eluent, to yield **1** (10.0 mg). The soluble in 50% MeOH in H_2_O part (77.0 mg) of fraction 383L was purified by reversed-phase HPLC, using MeCN/H_2_O (40:60) as eluent, to yield **6** (1.7 mg) and **13** (1.1 mg).

The bacterial strain BI0618 was streaked from a glycerol stock onto 25 freshly prepared agar plates containing a seawater-based (A1BFe+C) medium. After 3 days, when sufficient growth of the bacterial strain was observed, mycelia were picked from the agar plates and were inoculated into 1 L flasks containing 400 mL of the same seawater-based medium, to a total of 10 L of liquid medium, which were incubated at 24 °C for 8 days, while shaking at 130 rpm in an orbit shaker. At the end of the fermentation period, Amberlite XAD-7HP resin (20 g/L) was added to each flask to adsorb extracellular metabolites. The culture and resin were shaken overnight at low speed. The broth was centrifuged and the pellet (resin and cell mass) was extracted twice for 24 h with Me_2_CO (8 L in total). Filtration of the extract and removal of the solvent under vacuum at 38 °C afforded a solid residue, which was partitioned between EtOAc and H_2_O. Evaporation of the solvent of the EtOAc soluble fraction in vacuo afforded a dark orange oily residue (498 mg) that was subjected to vacuum column chromatography on silica gel, using cyclohexane, with increasing amounts of EtOAc, followed by EtOAc with increasing amounts of MeOH as the mobile phase, to yield 12 fractions (618A–618L). Fraction 618F (100% EtOAc, 8.4 mg) was identified as compound **4**. Fraction 618G (5% MeOH in EtOAc, 40.1 mg) was subjected to normal-phase HPLC, using cyclohexane/Me_2_CO (55:45) as eluent, to afford compounds **4** (6.1 mg) and **8** (5.3 mg). Fraction 618H (20% MeOH in EtOAc, 140.1 mg) was subjected to vacuum column chromatography on silica gel, using cyclohexane with increasing amounts of Me_2_CO, followed by Me_2_CO with increasing amounts of MeOH as the mobile phase, to afford 10 fractions (618H1-618H10). The soluble in 80% MeOH in H_2_O part (27.0 mg) of fraction 618H2 (60% Me_2_CO in cyclohexane) was purified by reversed-phase HPLC, using MeOH/H_2_O (60:40 and subsequently 50:50) as eluent, to yield **5** (0.3 mg), **11** (4.5 mg), **12** (0.4 mg), **14** (4.4 mg), **21** (1.1 mg), **26** (1.3 mg), **27** (0.9 mg), **28** (1.1 mg), and **32** (1.0 mg).

The bacterial strain BI0918 was inoculated from a glycerol stock into a 100 mL flask containing 50 mL of freshly prepared seawater-based (A1BFe+C) medium. After 4 days of incubation at 24 °C, while shaking at 120 rpm in an orbit shaker, the starter culture was streaked onto 25 freshly prepared agar plates containing the same seawater-based medium. After 7 days when sufficient growth of the bacterial strain was observed, mycelia were picked from the agar plates and were inoculated into 1 L flasks containing 400 mL of the same seawater-based medium, to a total of 20 L of liquid medium, which were incubated at 24 °C for 8 days, while shaking at 120 rpm in an orbit shaker. At the end of the fermentation period, Amberlite XAD-7HP resin (20 g/L) was added to each flask to adsorb extracellular metabolites. The culture and resin were shaken overnight at low speed. The broth was centrifuged and the pellet (resin and cell mass) was extracted twice for 24 h with Me_2_CO (12 L in total). Filtration of the extract and removal of the solvent under vacuum at 38 °C afforded a solid residue, which was partitioned between EtOAc and H_2_O. Evaporation of the solvent of the EtOAc soluble fraction in vacuo afforded a dark red oily residue (2.0 g) that was subjected to vacuum column chromatography on silica gel, using cyclohexane with increasing amounts of EtOAc, followed by EtOAc with increasing amounts of MeOH as the mobile phase, to yield 14 fractions (918A–918N). Fractions 918G (70% EtOAc in cyclohexane, 45.8 mg), 918H (80% EtOAc in cyclohexane, 52.0 mg), 918I (90% EtOAc in cyclohexane, 10.5 mg), and 918J (100% EtOAc and 5% MeOH in EtOAc, 59.8 mg) were separately purified by normal-phase HPLC, using cyclohexane/Me_2_CO (70:30 and/or 65:35) as eluent, to yield **4** (20.2 mg). Fraction 918K (10% MeOH in EtOAc, 114.7 mg) was further fractionated by vacuum column chromatography on silica gel C-18, using H_2_O with increasing amounts of MeOH as the mobile phase, to afford 5 fractions (918K1–918K5). Fractions 918K1 (20% MeOH in H_2_O, 41.5 mg) and 918K2 (40% MeOH in H_2_O, 28.1 mg) were separately purified by reversed-phase HPLC, using MeOH/H_2_O (30:70) and subsequently MeCN/H_2_O (30:70) as eluent, to yield **3** (7.0 mg), **4** (7.3 mg), **5** (2.0 mg), **8** (13.5 mg), and **11** (0.3 mg). Fraction 918K3 (60% MeOH in H_2_O, 14.4 mg) was purified by reversed-phase HPLC, using MeOH/H_2_O (50:50) as eluent, to yield **21** (1.1 mg), **22** (5.4 mg), and **23** (1.3 mg). Fraction 918L (25% MeOH in EtOAc, 113.5 mg) was further fractionated by vacuum column chromatography on silica gel C-18, using H_2_O with increasing amounts of MeOH as the mobile phase, to afford 5 fractions (918L1-918L5). Fraction 918L1 (20% MeOH in H_2_O, 22.3 mg) was purified by reversed-phase HPLC, using MeOH/H_2_O (30:70 and subsequently 25:75) as eluent, to yield **2** (0.5 mg), **14** (4.7 mg), **16** (0.4 mg), and **18** (0.4 mg). Fraction 918L2 (40% MeOH in H_2_O, 42.9 mg) was purified by reversed-phase HPLC, using MeOH/H_2_O (50:50) and subsequently MeCN/H_2_O (30:70) as eluent, and finally normal-phase HPLC, using cyclohexane/Me_2_CO (20:80) as eluent, to yield **5** (1.1 mg), **9** (0.9 mg), **11** (10.9 mg), **12** (3.3 mg), **14** (2.0 mg), **17** (2.0 mg), **19** (0.5 mg), **20** (1.1 mg), **21** (0.6 mg), and **26** (2.2 mg). Fraction 918L3 (60% MeOH in H_2_O, 10.7 mg) was purified by reversed-phase HPLC, using MeOH/H_2_O (50:50) as eluent and subsequently normal-phase HPLC, using cyclohexane/Me_2_CO (30:70) as eluent, to yield **26** (2.0 mg), **27** (2.0 mg), **28** (2.0 mg), and **32** (2.4 mg). Fractions 918M (100% MeOH, 464.0 mg) and 918N (100% MeOH, 23.0 mg) were combined and fractionated by vacuum column chromatography on silica gel C-18, using H_2_O with increasing amounts of MeOH as the mobile phase, to afford 5 fractions (918M1-918M5). Fractions 918M1 (20% MeOH in H_2_O, 186.2 mg), 918M2 (40% MeOH in H_2_O, 30.9 mg), and 918M3 (60% MeOH in H_2_O, 12.9 mg) were separately and repeatedly purified by reversed-phase HPLC, using MeOH/H_2_O (40:60 and subsequently 25:75) as eluent, to yield **10** (0.5 mg) and **24** (4.1 mg).

The bacterial strain BI0980 was inoculated from a glycerol stock into two 100 mL flasks containing 50 mL of freshly prepared seawater-based (A1BFe+C) medium. After 5 days of incubation at 24 °C while shaking at 120 rpm in an orbit shaker, the starter cultures were inoculated into two 1 L flasks containing 500 mL of the same seawater-based medium (10% *v*/*v* inoculum) that were incubated at 24 °C for 4 days, while shaking at 120 rpm in an orbit shaker. Subsequently, they were inoculated into 1 L flasks containing 500 mL of the same seawater-based medium (10% *v*/*v* inoculum), to a total of 10 L of liquid medium, that were incubated at 24 °C for 9 days while shaking at 120 rpm in an orbit shaker. At the end of the fermentation period, Amberlite XAD-7HP resin (20 g/L) was added to each flask to adsorb extracellular metabolites. The culture and resin were shaken overnight at low speed. The broth was centrifuged and the pellet (resin and cell mass) was extracted twice for 24 h with Me_2_CO (6 L in total). Filtration of the extract and removal of the solvent under vacuum at 38 °C afforded a solid residue, which was partitioned between EtOAc and H_2_O. Evaporation of the solvent of the EtOAc soluble fraction in vacuo afforded a dark red oily residue (533.9 mg) that was subjected to vacuum column chromatography on silica gel, using cyclohexane with increasing amounts of EtOAc, followed by EtOAc with increasing amounts of MeOH as the mobile phase, to yield 14 fractions (980A–980N). Fraction 980J (5% MeOH in EtOAc, 26.8 mg) was purified by normal-phase HPLC, using cyclohexane/Me_2_CO (50:50) as eluent, to yield **4** (11.2 mg) and **8** (3.3 mg). Fraction 980K (10% MeOH in EtOAc, 51.8 mg) was repeatedly purified by normal-phase HPLC, using cyclohexane/acetone (30:70 and 20:80) as eluent, and reversed-phase HPLC, using MeCN/H_2_O (20:80 and 30:70) as eluent, to yield **3** (4.4 mg), **4** (3.2 mg), **5** (4.1 mg), **8** (6.9 mg), **9** (2.1 mg), **11** (6.3 mg), **12** (0.9 mg), **14** (4.3 mg), **15** (1.6 mg), **19** (2.8 mg), **20** (0.3 mg), **21** (1.3 mg), **26** (2.9 mg), and a mixture (1:1) of **30,** and **31** (0.4 mg). Fraction 980L (25% MeOH in EtOAc, 88.3 mg) was further fractionated by vacuum column chromatography on silica gel C-18, using H_2_O with increasing amounts of MeOH as the mobile phase, to afford 3 fractions (980L1-980L3). Fractions 980L1 (20% MeOH in H_2_O, 32.3 mg) and 980L2 (40% to 60% MeOH in H_2_O, 11.8 mg) were combined and purified by reversed-phase HPLC, using MeOH/H_2_O (30:70) as eluent, and subsequently normal-phase HPLC, using cyclohexane/Me_2_CO (20:80) as eluent, to yield **12** (1.0 mg), **16** (0.9 mg), **20** (1.9 mg), **24** (0.8 mg), **25** (0.5 mg), and **29** (1.7 mg).

*cis*-Cyclo(Pro-3-chloro-Tyr) (**15**): white solid; ${\left[\alpha \right]}_{D}^{\mathrm{20}}$ +69.0 (*c* 0.021, CHCl_3_); UV (CHCl_3_) *λ*_max_ (log *ε*) 240 (2.68), 280 (2.90) nm; IR (thin film) *ν*_max_ 3231, 2928, 1651, 1457, 1295 cm^−1^; ^1^H NMR data, see Table 1; ^13^C NMR data, see Table 2; HRESIMS *m*/*z* 317.0661/319.0629 (3:1) [M + Na]^+^ (calcd. for C_14_H_15_N_2_O_3_^35^ClNa, 317.0663, C_14_H_15_N_2_O_3_^37^ClNa, 319.0634).

*trans*-Cyclo(Pro-3-chloro-Tyr) (**16**): white solid; ${\left[\alpha \right]}_{D}^{\mathrm{20}}$ +87.0 (*c* 0.023, CHCl_3_); UV (CHCl_3_) *λ*_max_ (log *ε*) 240 (2.79), 280 (2.97) nm; IR (thin film) *ν*_max_ 3235, 2929, 1650, 1455, 1293 cm^−1^; ^1^H NMR data, see Table 1; ^13^C NMR data, see Table 2; HRESIMS *m*/*z* 317.0657/319.0627 (3:1) [M + Na]^+^ (calcd. for C_14_H_15_N_2_O_3_^35^ClNa, 317.0663, C_14_H_15_N_2_O_3_^37^ClNa, 319.0634).

*cis*-Cyclo(3-chloro-Tyr-Ile) (**31**): white solid; ^1^H NMR data, see Table 1; ^13^C NMR data, see Table 2; EIMS *m*/*z* 310/312 (3:1) [M]^+^ (calcd. for C_15_H_19_N_2_O_3_Cl, 310/312).

## 4. Conclusions

The chemical investigation of the organic extracts of the fermentation broths of five marine-derived strains isolated from sediments collected from the East Mediterranean Sea resulted in the isolation and structure elucidation of three new 2,5-DKPs, namely *cis*-cyclo(Pro-3-chloro-Tyr) (**15**), *trans*-cyclo(Pro-3-chloro-Tyr) (**16**), and *cis*-cyclo(3-chloro-Tyr-Ile) (**31**). It is not unusual for marine macro- and microorganisms to incorporate halogens, mainly chlorine and bromine atoms, in their secondary metabolism, in order to increase the bioactivity of the compounds they biosynthesize [57,58]. Indeed, the brominated analogues of **15** and **16** have already been isolated from the actinobacterium *Nocardia ignorata* [59]. Additionally, the relevant literature is supplemented with complete NMR assignments and revisions for 29 previously reported 2,5-DKPs.

## Figures and Tables

**Table:** (**a**)

Position	1 ^2^	2 ^2^	3 ^2^	4 ^2^	5 ^2^	6 ^2^	7 ^2^	8 ^2^
1	-	-	-	-	-	-	-	-
3	4.08, d (16.6) 3.87, dd (16.6, 4.4)	4.12, m	3.92, br s	4.00, dd (9.7, 3.6)	3.91, ddd (9.7, 5.5, 4.4)	4.04, dd (9.6, 3.8)	3.95, m	3.95, br s
4	6.32, br s	5.66, br s	5.67, br s	5.78, br s	6.04, br s	5.83, br s	6.07, br s	5.86, br s
6	4.07, m	4.11, m	4.07, t (8.1)	4.10, t (8.2)	4.07, dd (9.4, 6.8)	4.48, dd (11.1, 6.3)	4.16, dd (9.2, 5.6)	4.05, br t (8.0)
7	2.36, m	2.36, m	2.37, m	2.33, m	2.38, m	2.37, br dd (13.5, 6.3)	2.48, m	2.36, m
	2.05, m	2.13, m	2.02, m	2.12, m	2.02, m	2.14, ddd (13.5, 11.1, 4.3)		2.02, m
8	2.00, m	2.01, m	2.02, m	2.01, m	2.02, m	4.58 (t, 4.3)	4.46, m	2.02, m
	1.90, m	1.89, m	1.89, m	1.89, m	1.88, m			1.89, m
9	3.61, m 3.53, m	3.56, m	3.62, m 3.53, m	3.55, m	3.63, m 3.51, m	3.71, dd (13.2, 4.3) 3.55, d (13.2)	3.95, m 3.34, dd (12.5, 4.8)	3.61, m 3.53, m
10		1.45, d (7.0)	2.62, qqd (7.3, 6.9, 2.7)	2.05, m 1.51, ddd (14.5, 9.7, 5.0)	1.62, m	2.05, ddd (14.5, 9.9, 3.8) 1.50, ddd (14.5, 9.6, 4.9)	1.62, m	2.30, m
11			0.89, d (6.9)	1.71, m	1.75, m	1.73, m	1.74, m	1.41, m 1.14, m
12			1.04, d (7.3)	0.94, d (6.5)	0.93, d (6.5)	0.94, d (6.6)	0.93, d (6.5)	0.91, t (7.4)
13				0.98, d (6.5)	0.97, d (6.5)	0.99, d (6.6)	0.96, d (6.5)	1.03, d (7.2)
14								
15								
16								
17								
18								
19								
20								
OH								

^1^ In CDCl_3_ for **1**–**28** and **30**–**32** and in CD_3_OD for **29**. ^2^ Recorded at 400 MHz. ^3^ Recorded at 950 MHz. ^4^ Recorded at 700 MHz. ^5^ Recorded at 600 MHz.

**Table:** (**b**)

Position	9 ^2^	10 ^2^	11 ^2^	12 ^2^	13 ^2^	14 ^2^	15 ^3^	16 ^3^
1	-	-	-	-	-	-	-	-
3	3.77, dd (5.4, 3.9)	4.19, m	4.25, dd (10.5, 3.5)	4.19, dt (6.7, 4.3)	4.30, dd (10.8, 3.6)	4.20, dd (10.1, 3.5)	4.21, td (10.0, 3.3)	4.13, ddd (7.0, 4.2, 3.5)
4	6.19, br s	7.43, s	5.69, br s	5.76, br s	5.57, br s	5.85, br s	5.55, br s	5.75, br s
6	4.08, dd (9.6, 6.5)	4.10, m	4.05, t (8.0)	3.04, dd (10.4, 6.6)	4.45, dd (11.2, 6.4)	4.07, t (7.9)	4.07, m	3.22, dd (10.8, 6.6)
7	2.39, m 1.93, m	2.36, m 2.14, m	2.31,m 2.02, m	2.19, m 1.79, m	2.35, dd (13.5, 6.4) 2.06, ddd (13.5, 11.2, 4.3)	2.32, m 1.94, m	2.33, m 2.00, m	2.25, dt (12.0, 6.6) 1.84, ddd (12.0, 10.8, 7.5)
8	2.01, m 1.87,m	2.02, m 1.89, m	2.02, m 1.88, m	1.94, m 1.69, m	4.59, t (4.3)	2.07, m .87, m	2.00, m 1.89, m	1.96, m 1.74, m
9	3.68, m 3.50, m	3.55, m	3.62, m 3.59, m	3.62, ddd (12.0, 9.4, 8.4) 3.40, ddd (12.0, 8.4, 3.0)	3.78, dd (13.4, 4.3) 3.57, d (13.4)	3.63, m 3.55, m	3.62, dt (11.5, 8.0) 3.55, ddd (11.5, 9.0, 2.4)	3.64, dt (11.9, 8.4) 3.42, ddd (11.9, 9.3, 2.8)
10	1.93, m	2.50, m 2.36, m	3.62, m 2.76, dd (14.5, 10.5)	3.08, m	3.62, dd (14.5, 3.6) 2.75, dd (14.5, 10.8)	3.46, dd (14.6, 3.5) 2.74, dd (14.6, 10.1)	3.48, dd (14.8, 3.3) 2.73, dd (14.8, 10.0)	3.01, dd (14.0, 7.0) 2.98, dd (14.0, 4.2)
11	1.54, m 1.22, m	3.02, m 2.79, m	-	-	-	-	-	-
12	0.91, t (7.4)	2.59, s	7.20, br d (7.2)	7.19, br d (7.2)	7.21, br d (7.4)	7.03, d (8.6)	7.18, d (2.0)	7.17, d (2.0)
13	1.00, d (6.9)		7.33, br t (7.2)	7.30, br t (7.2)	7.34, br t (7.4)	6.76, d (8.6)	-	-
14			7.28, br t (7.2)	7.28, br t (7.2)	7.29, br t (7.4)	-	-	-
15			7.33, br t (7.2)	7.30, br t (7.2)	7.34, br t (7.4)	6.76, d (8.6)	6.98, d (8.2)	6.95, d (8.3)
16			7.20, br d (7.2)	7.19, br d (7.2)	7.21, br d (7.4)	7.03, d (8.6)	7.03, dd (8.2, 2.0)	7.00, dd (8.3, 2.0)
17								
18								
19								
20								
OH						6.69, br s	5.55, br s	5.53, br s

^1^ In CDCl_3_ for **1**–**28** and **30**–**32** and in CD_3_OD for **29**. ^2^ Recorded at 400 MHz. ^3^ Recorded at 950 MHz. ^4^ Recorded at 700 MHz. ^5^ Recorded at 600 MHz.

**Table:** (**c**)

Position	17 ^2^	18 ^4^	19 ^2^	20 ^5^	21 ^2^	22 ^5^	23 ^2^	24 ^5^
1	-	5.92, br s	5.92, br s	5.88, br s	6.27, br s	6.13, br s	5.97, br s	5.73, br s
3	4.36, dd (10.9, 3.0)	4.02, d (17.4) 3.98, d (17.4)	4.09, q (7.0)	4.11, q (7.0)	3.89, br s	3.97, br d (9.9)	3.94, br s	4.00, q (7.0)
4	5.72, br s	5.76, br s	5.89, br s	5.83, br s	6.10, br s	6.13, br s	5.84, br s	5.79, br s
6	4.06, t (8.0)	3.97, m	3.99, m	3.95, br s	4.00, br d (10.1)	3.97, br d (9.9)	4.00, br d (10.2)	4.26, br d (8.2)
7	2.31, m 2.00, m	1.82, ddd (13.3, 9.6, 4.3) 1.66, ddd (13.3, 9.7, 4.5)	1.89, ddd (13.9, 9.5, 3.8) 1.62, ddd (13.9, 9.7, 4.8)	2.12, m	1.88, ddd (13.7, 9.8, 3.7) 1.60, ddd (13.7, 10.1, 4.6)	1.85, ddd (13.4, 9.6, 3.7) 1.61, m	1.89, ddd (13.6, 9.9, 3.7) 1.60, m	3.31, dd (14.0, 3.6) 3.00, dd (14.0, 8.2)
8	2.00, m 1.89, m	1.76, m	1.75, m	1.46, m 1.25, m	1.76, m	1.76, m	1.76, m	-
9	3.60, m	0.94, d (6.4)	0.94, d (6.5)	0.93, t (7.5)	0.93, d (6.9)	0.93, d (6.4)	0.93, d (6.7)	7.21, br d (7.6)
10	3.75, dd (15.1, 3.6) 2.95, dd (15.1, 10.9)	0.99, d (6.4)	0.98, d (6.5)	1.01, d (7.3)	0.97, d (6.5)	0.98, d (6.4)	0.98, d (6.5)	7.32, br t (7.6)
11	-		1.50, d (7.0)	1.50, d (7.0)	2.41, qqd (7.0, 6.9, 3.4)	1.85, ddd (13.4, 9.6, 3.7) 1.61, m	2.10, m	7.28, br t (7.6)
12	7.10, br s				0.93, d (6.9)	1.76, m	1.45, m 1.22, m	7.32, br t (7.6)
13	8.20, br s				1.03, d (7.0)	0.93, d (6.4)	0.92, t (7.5)	7.21, br d (7.6)
14	-					0.98, d (6.4)	1.01, d (7.1)	1.14, d (7.0)
15	7.38, br d (8.0)							
16	7.22, br t (8.0)							
17	7.13, br t (8.0)							
18	7.57, br d (8.0)							
19	-							
20								
OH								

^1^ In CDCl_3_ for **1**–**28** and **30**–**32** and in CD_3_OD for **29**. ^2^ Recorded at 400 MHz. ^3^ Recorded at 950 MHz. ^4^ Recorded at 700 MHz. ^5^ Recorded at 600 MHz.

**Table:** (**d**)

Position	25 ^3^	26 ^2^	27 ^2^	28 ^5^	29 ^2^	30 ^4^	31 ^4^	32 ^5^
1	5.94, br s	6.10, br s	5.79, br s	5.78, br s	-	5.65, br s	5.65, br s	5.79, br s
3	4.03, dd (5.2, 4.6)	3.87, br s	3.86, br d (10.2)	3.91, br s	3.65, dd (10.0, 4.3)	3.90, br s	3.90, br s	4.13, br d (8.5)
4	5.71, br s	5.89, br s	5.90, br s	5.90, br s	-	5.73, br s	5.73, br s	5.79, br s
6	4.25, dd (8.9, 3.6)	4.22, br d (9.7)	4.26, dd (7.9, 3.9)	4.22, br d (9.6)	4.23, dd (4.6, 3.6)	4.15, br d (9.4)	4.19, br d (8.9)	4.13, br d (8.5)
7	3.35, dd (14.0, 3.6)	3.44, dd (13.8, 3.5)	3.23, dd (13.8, 3.9)	3.42, dd (13.8, 3.5)	3.20, dd (13.8, 3.6)	3.32, dd (14.0, 3.2)	3.24, dd (14.0, 3.2)	3.08, dd (13.8, 3.0
	3.02, dd (14.0, 8.9)	2.87, dd (13.8, 9.7)	3.05, dd (13.8, 7.9)	2.89,dd (13.8, 9.6)	2.82, dd (13.8, 4.6)	2.83, dd (14.0, 9.4)	2.89, dd (14.0, 8.9)	)2.30, dd (13.8, 8.5)
8	-	-	-	-	-	-	-	-
9	7.20, br d (7.4)	7.20, br d (7.1)	7.20, br d (7.3)	7.20, br d (7.1)	6.99, d (8.5)	7.07, d (8.4)	7.17, br s	7.11, br d (7.2)
10	7.34, br t (7.4)	7.33, br t (7.1)	7.33, br t (7.3)	7.33, br t (7.1)	6.71, d (8.5)	6.79, d (8.4)	-	7.34, br t (7.2)
11	7.29, br t (7.4)	7.27, br t (7.1)	7.28, br t (7.3)	7.28, br t (7.1)	-	-	-	7.28, br t (7.2)
12	7.34, br t (7.4)	7.33, br t (7.1)	7.33, br t (7.3)	7.33, br t (7.1)	6.71, d (8.5)	6.79, d (8.4)	6.97, d (8.3)	7.34, br t (7.2)
13	7.20, br d (7.4)	7.20, br d (7.1)	7.20, br d (7.3)	7.20, br d (7.1)	6.99, d (8.5)	7.07, d (8.4)	7.02, br d (8.3)	7.11, br d (7.2)
14	3.65, dd (11.0, 4.6) 3.42, dd (11.0, 5.2)	2.32, qqd (7.0, 7.0, 3.2)	1.57, m 0.82, m	2.00, m	0.89, m 0.10, ddd (14.7, 10.0, 4.9)	1.99, m	1.99, m	3.08, dd (13.8, 3.0) 2.30, dd (13.8, 8.5)
15		0.80, d (7.0)	1.49, m	1.26, m 1.05, m	1.44, m	1.19, m 1.00, m	1.30, m 1.03, m	-
16		0.99, d (7.0)	0.84, d (6.8)	0.87,t (7.4)	0.73, d (6.7)	0.85, t (7.5)	0.87, t (7.4)	7.11, br d (7.2)
17			0.85, d (6.8)	0.96, d (7.1)	0.75, d (6.7)	0.94, d (7.2)	0.96, d (7.2)	7.34, br t (7.2)
18								7.28, br t (7.2)
19								7.34, br t (7.2)
20								7.11, br d (7.2)
OH								

^1^ In CDCl_3_ for **1**–**28** and **30**–**32** and in CD_3_OD for **29**. ^2^ Recorded at 400 MHz. ^3^ Recorded at 950 MHz. ^4^ Recorded at 700 MHz. ^5^ Recorded at 600 MHz.

**Table:** (**a**)

Position	1 ^2^	2 ^2^	3 ^3,4^	4 ^4^	5 ^2^	6 ^3,4^	7 ^2^	8 ^3,4^	9 ^2^	10 ^2^	11 ^3,4^	12 ^2^	13 ^2^	14 ^4^	15 ^3,5^	16 ^3,5^
2	163.3	166.2	165.7	166.1	166.4	167.4	167.7	165.1	165.3	165.2	165.0	164.8	166.2	165.3	164.7	164.4
3	46.7	51.3	60.4	53.4	56.4	53.4	56.1	60.6	63.0	54.1	56.2	59.0	56.2	56.3	55.8	58.9
5	169.5	170.0	170.1	170.2	169.6	170.0	169.8	169.9	169.4	170.2	169.4	169.3	169.8	169.7	169.1	168.4
6	58.5	59.4	58.8	59.0	58.1	57.3	56.2	58.9	58.4	59.6	59.1	57.7	57.4	59.1	59.0	57.6
7	28.5	28.2	28.6	28.1	29.1	37.8	36.8	28.6	29.5	28.2	28.3	28.9	37.7	28.2	28.2	28.7
8	22.4	22.8	22.4	23.3	23.1	68.6	68.1	22.4	22.1	22.8	22.5	21.7	68.2	22.3	22.4	21.5
9	45.4	45.5	45.2	45.5	45.7	54.5	54.0	45.2	45.7	45.5	45.5	45.1	54.4	45.3	45.3	45.1
10		16.2	28.4	38.6	42.6	38.5	42.2	35.3	39.7	23.2	36.8	40.5	36.7	35.9	35.5	39.2
11			16.1	24.7	24.5	24.9	24.6	24.1	24.6	49.4	135.9	135.3	135.8	126.5	128.8	128.4
12			19.2	21.2	21.4	21.7	21.5	12.2	11.4	38.5	129.3	129.9	129.3	130.4	129.3	129.7
13				22.7	22.3	23.6	23.0	16.0	15.4		129.1	128.8	129.2	116.0	120.2	120.0
14											127.6	127.3	127.6	155.9	150.6	150.8
15											129.1	128.8	129.2	116.0	116.7	116.3
16											129.3	129.9	129.3	130.4	129.0	129.5
17																
18																
19																
20																

^1^ In CDCl_3_ for **1**–**28** and **30**–**32** and in CD_3_OD for **29**. ^2^ Adapted (and revised when appropriate) as follows: **1** [25], **2** [27], **5** [31], **7** [35], **9** [37], **10** [38], **12** [40], **13** [41], **17** [44], **21** [37], **23** [17], **27** [17], **29** [54]. ^3^ Determined through HMBC correlations. ^4^ Recorded at 100 MHz. ^5^ Recorded at 237.5 MHz. ^6^ Recorded at 175 MHz. ^7^ Recorded at 150 MHz. ^8^ nd: not detected.

**Table:** (**b**)

Position	17 ^2^	18 ^3,6^	19 ^3,4^	20 ^3,7^	21 ^2^	22 ^3,7^	23 ^2^	24 ^3,7^	25 ^3,5^	26 ^3,4^	27 ^2^	28 ^3,7^	29 ^2^	30 ^3,6^	31 ^3,6^	32 ^3,7^
2	165.7	165.3	nd ^8^	nd ^8^	167.4	nd ^8^	167.3	nd ^8^	nd ^8^	nd ^8^	167.6	nd ^8^	167.6	166.1	166.2	nd ^8^
3	54.7	44.6	51.1	50.6	60.3	53.0	60.1	50.7	55.8	60.4	53.4	59.8	52.8	59.9	59.9	56.1
5	169.5	168.1	nd ^8^	nd ^8^	169.0	nd ^8^	168.8	nd ^8^	nd ^8^	nd ^8^	167.8	nd ^8^	171.4	nd ^8^	nd ^8^	nd ^8^
6	59.3	53.2	53.7	60.0	53.2	53.0	53.2	56.2	55.8	56.5	56.4	55.9	56.3	55.8	55.9	56.1
7	28.4	42.3	42.7	37.7	43.9	42.7	43.6	39.8	39.8	41.0	40.2	40.2	38.1	39.3	38.9	40.1
8	22.7	24.0	25.2	23.6	24.3	24.3	24.1	135.3	134.9	135.4	135.1	135.6	125.7	127.3	128.2	135.3
9	45.5	21.0	21.7	11.5	21.1	20.8	21.2	129.6	129.3	129.6	130.2	129.3	131.4	130.6	129.7	129.5
10	26.9	22.8	23.6	15.0	23.4	23.0	23.5	128.8	128.9	129.0	129.2	128.9	115.1	115.7	120.1	128.7
11	109.3		20.4	20.5	31.6	42.7	38.3	127.5	127.4	127.7	127.8	127.3	157.0	155.0	150.4	127.4
12	123.6				16.5	24.3	24.4	128.8	128.9	129.0	129.2	128.9	115.1	115.7	116.6	128.7
13	-				18.9	20.8	11.9	129.6	129.3	129.6	130.2	129.3	131.4	130.6	129.5	129.5
14	136.8					23.0	15.4	19.8	63.7	31.6	43.1	37.9	43.9	37.8	37.8	40.1
15	111.7									16.4	23.2	23.3	23.3	23.3	23.1	135.3
16	122.8									19.3	20.9	11.3	20.0	11.5	11.5	129.5
17	120.0										22.8	15.1	22.1	15.1	15.1	128.7
18	118.6															127.4
19	126.8															128.7
20																129.5

^1^ In CDCl_3_ for **1**–**28** and **30**–**32** and in CD_3_OD for **29**. ^2^ Adapted (and revised when appropriate) as follows: **1** [25], **2** [27], **5** [31], **7** [35], **9** [37], **10** [38], **12** [40], **13** [41], **17** [44], **21** [37], **23** [17], **27** [17], **29** [54]. ^3^ Determined through HMBC correlations. ^4^ Recorded at 100 MHz. ^5^ Recorded at 237.5 MHz. ^6^ Recorded at 175 MHz. ^7^ Recorded at 150 MHz. ^8^ nd: not detected.

## Supplementary Materials

The following are available online at, Figures S1–S17: 1D and 2D NMR and MS spectra of compounds **15**, **16** & **31**.
