# Supplementary material for: New Chlorinated 2,5-Diketopiperazines from Marine-Derived Bacteria Isolated from Sediments of the Eastern Mediterranean Sea
Source: Molecules. 2020 Mar 26;25(7):1509. doi: 10.3390/molecules25071509 (PMC7181205; doi:10.3390/molecules25071509)
Supplement: Supplementary file 1 [file molecules-25-01509-s001.pdf]

Supplementary Materials for

New Chlorinated 2,5-Diketopiperazines from Marine-Derived Bacteria Isolated from Sediments of the Eastern Mediterranean Sea

Maria Harizani [1], Eleni Katsini [1], Panagiota Georgantea [1], Vassilios Roussis [1] and Efstathia Ioannou [1,*]

[1] Section of Pharmacognosy and Chemistry of Natural Products, Department of Pharmacy, School of Health Sciences, National and Kapodistrian University of Athens, Panepistimiopolis Zografou, Athens 15771, Greece; mariachariz@pharm.uoa.gr (M.H.); ekatsini@pharm.uoa.gr (E.K.); ggeorgantea@yahoo.gr (P.G.); roussis@pharm.uoa.gr (V.R.)

* Correspondence: eioannou@pharm.uoa.gr; Tel.: +30 210 727 4913

Table of Contents

**Figure S1.** ^{1}H NMR spectrum of *cis*-cyclo(Pro-3-chloro-Tyr) (**15**) in $CDCl_3$.	S3
**Figure S2.** HSQC spectrum of *cis*-cyclo(Pro-3-chloro-Tyr) (**15**) in $CDCl_3$.	S3
**Figure S3.** HMBC spectrum of *cis*-cyclo(Pro-3-chloro-Tyr) (**15**) in $CDCl_3$.	S4
**Figure S4.** COSY spectrum of *cis*-cyclo(Pro-3-chloro-Tyr) (**15**) in $CDCl_3$.	S4
**Figure S5.** NOESY spectrum of *cis*-cyclo(Pro-3-chloro-Tyr) (**15**) in $CDCl_3$.	S5
**Figure S6.** HRESIMS spectrum of *cis*-cyclo(Pro-3-chloro-Tyr) (**15**).	S5
**Figure S7.** ^{1}H NMR spectrum of *trans*-cyclo(Pro-3-chloro-Tyr) (**16**) in $CDCl_3$.	S6
**Figure S8.** HSQC spectrum of *trans*-cyclo(Pro-3-chloro-Tyr) (**16**) in $CDCl_3$.	S6
**Figure S9.** HMBC spectrum of *trans*-cyclo(Pro-3-chloro-Tyr) (**16**) in $CDCl_3$.	S7
**Figure S10.** COSY spectrum of *trans*-cyclo(Pro-3-chloro-Tyr) (**16**) in $CDCl_3$.	S7
**Figure S11.** NOESY spectrum of *trans*-cyclo(Pro-3-chloro-Tyr) (**16**) in $CDCl_3$.	S8
**Figure S12.** HRESIMS spectrum of *trans*-cyclo(Pro-3-chloro-Tyr) (**16**).	S8
**Figure S13.** ^{1}H NMR spectrum of the 1:1 mixture of *cis*-cyclo(Tyr-Ile) (**30**) and *cis*-cyclo(3-chloro-Tyr-Ile) (**31**) in $CDCl_3$.	S9
**Figure S14.** HSQC spectrum of the 1:1 mixture of *cis*-cyclo(Tyr-Ile) (**30**) and *cis*-cyclo(3-chloro-Tyr-Ile) (**31**) in $CDCl_3$.	S9
**Figure S15.** HMBC spectrum of the 1:1 mixture of *cis*-cyclo(Tyr-Ile) (**30**) and *cis*-cyclo(3-chloro-Tyr-Ile) (**31**) in $CDCl_3$.	S10
**Figure S16.** COSY spectrum of the 1:1 mixture of *cis*-cyclo(Tyr-Ile) (**30**) and *cis*-cyclo(3-chloro-Tyr-Ile) (**31**) in $CDCl_3$.	S10
**Figure S17.** EIMS spectrum of *cis*-cyclo(3-chloro-Tyr-Ile) (**31**).	S11

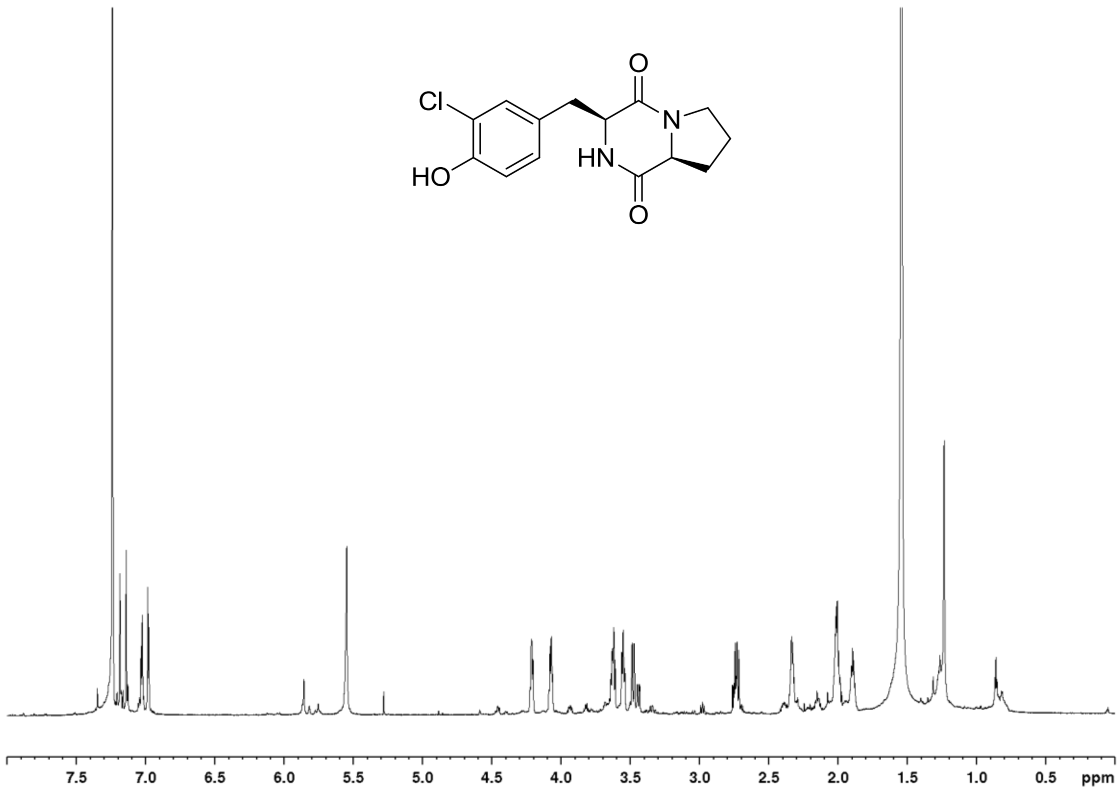

Figure S1. ^{1}H NMR spectrum of *cis*-cyclo(Pro-3-chloro-Tyr) (**15**) in $CDCl_3$.

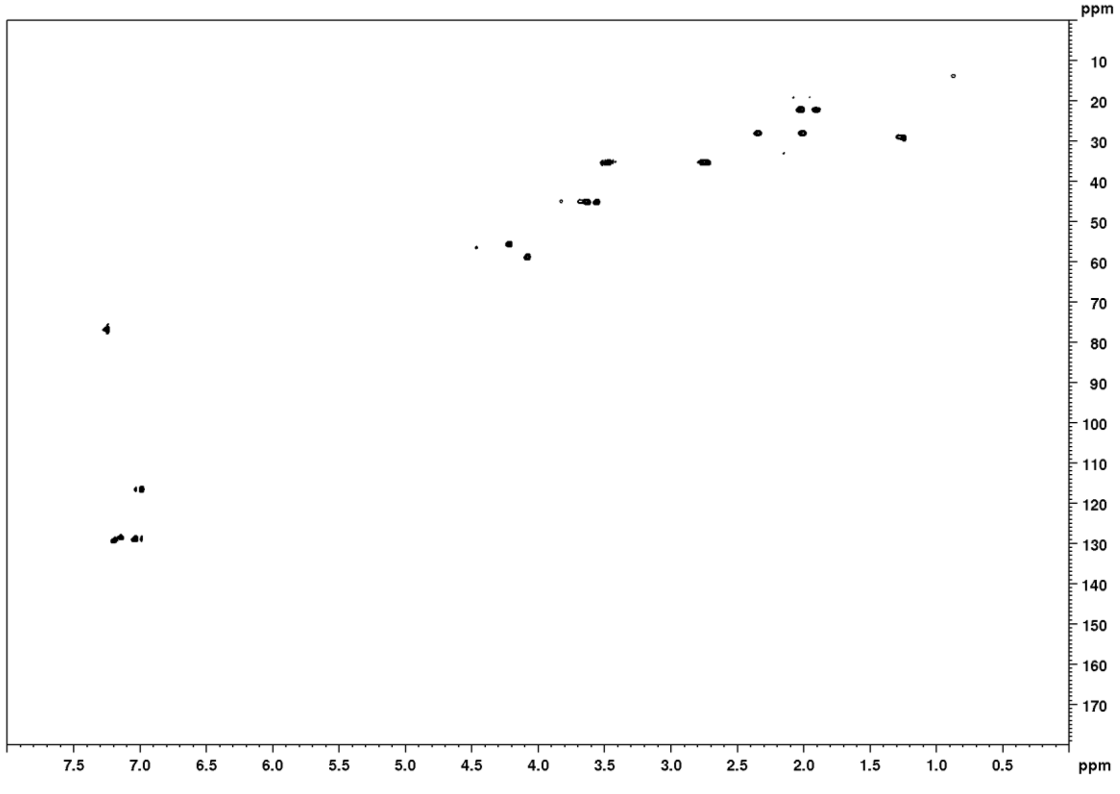

Figure S2. HSQC spectrum of *cis*-cyclo(Pro-3-chloro-Tyr) (**15**) in $CDCl_3$.

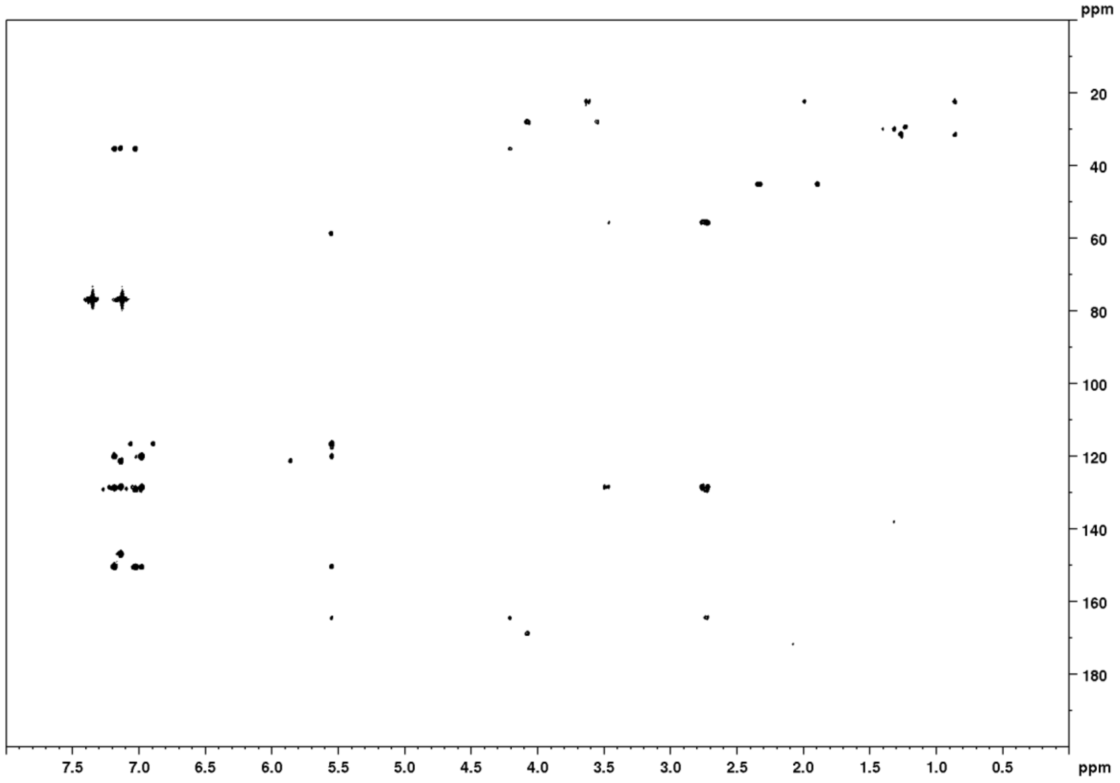

Figure S3. HMBC spectrum of *cis*-cyclo(Pro-3-chloro-Tyr) (**15**) in $CDCl_3$.

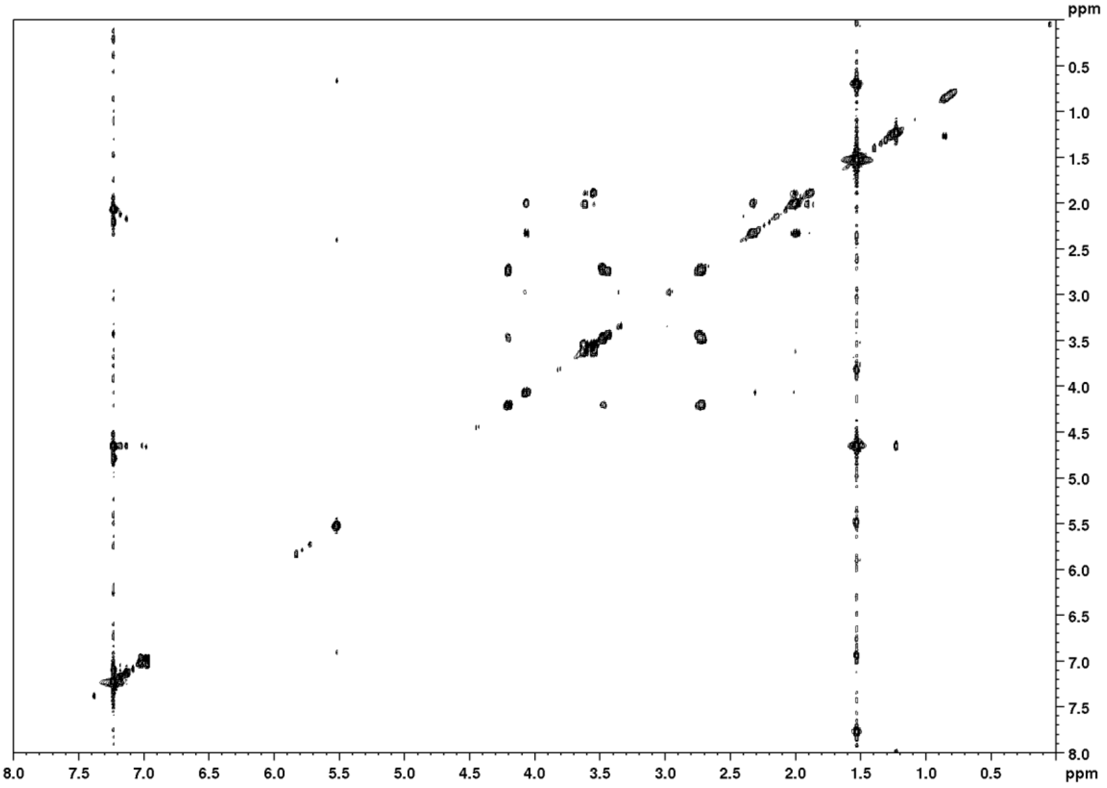

Figure S4. COSY spectrum of *cis*-cyclo(Pro-3-chloro-Tyr) (**15**) in $CDCl_3$.

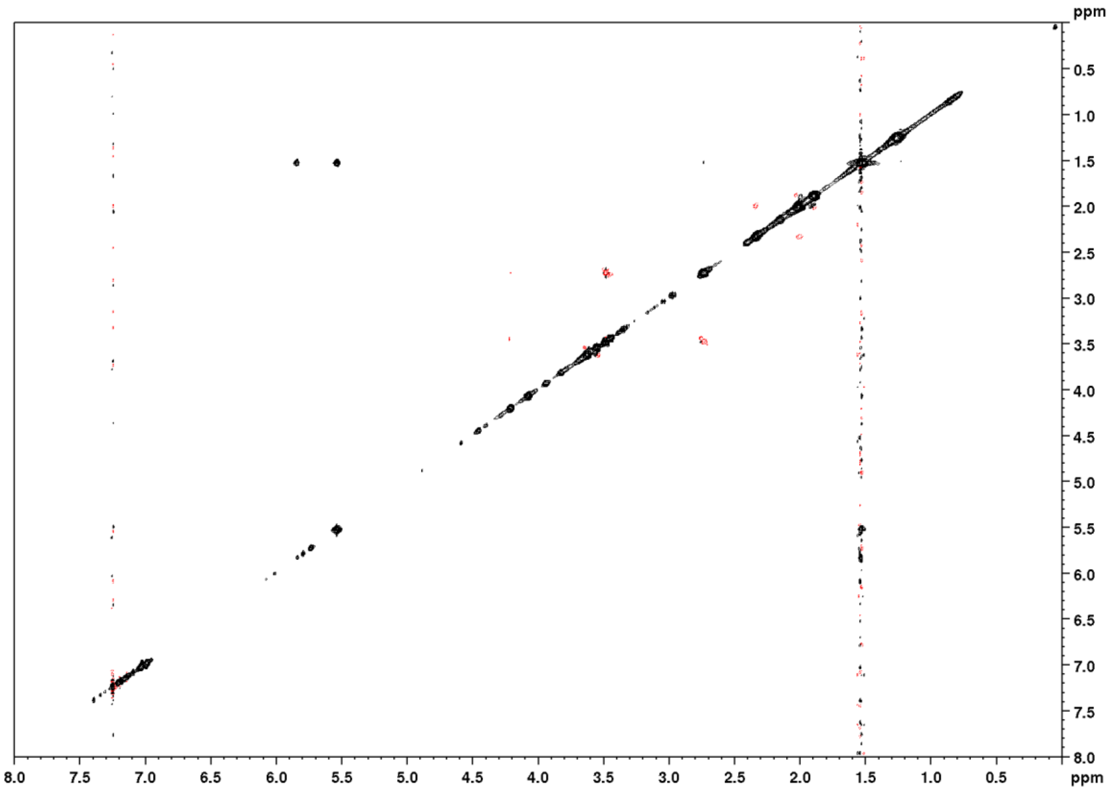

Figure S5. NOESY spectrum of *cis*-cyclo(Pro-3-chloro-Tyr) (**15**) in $CDCl_3$.

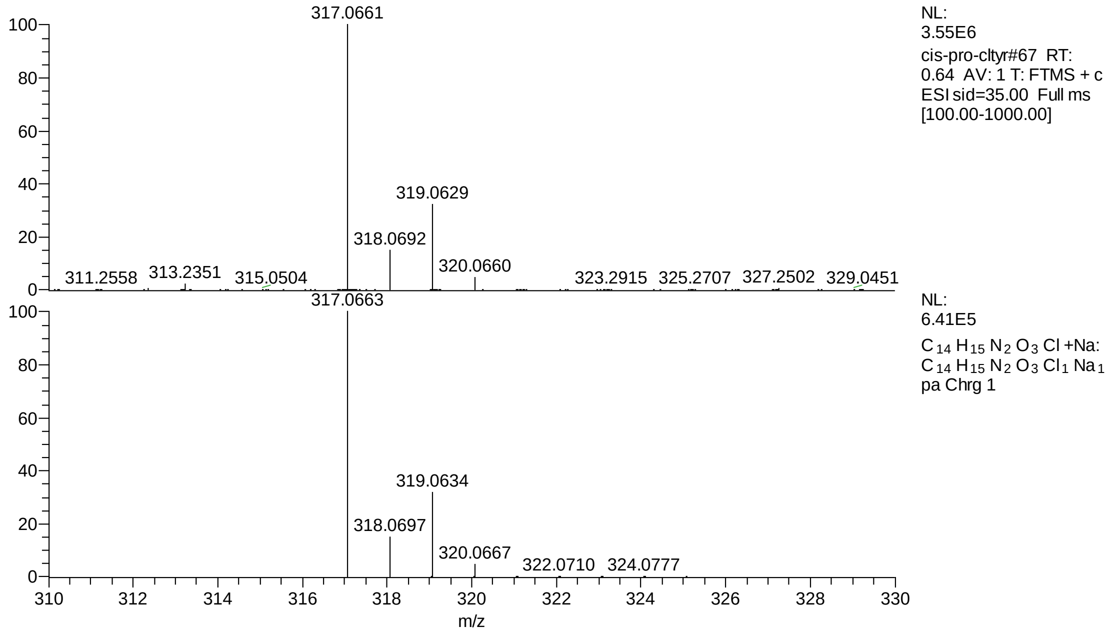

Figure S6. HRESIMS spectrum of *cis*-cyclo(Pro-3-chloro-Tyr) (**15**).

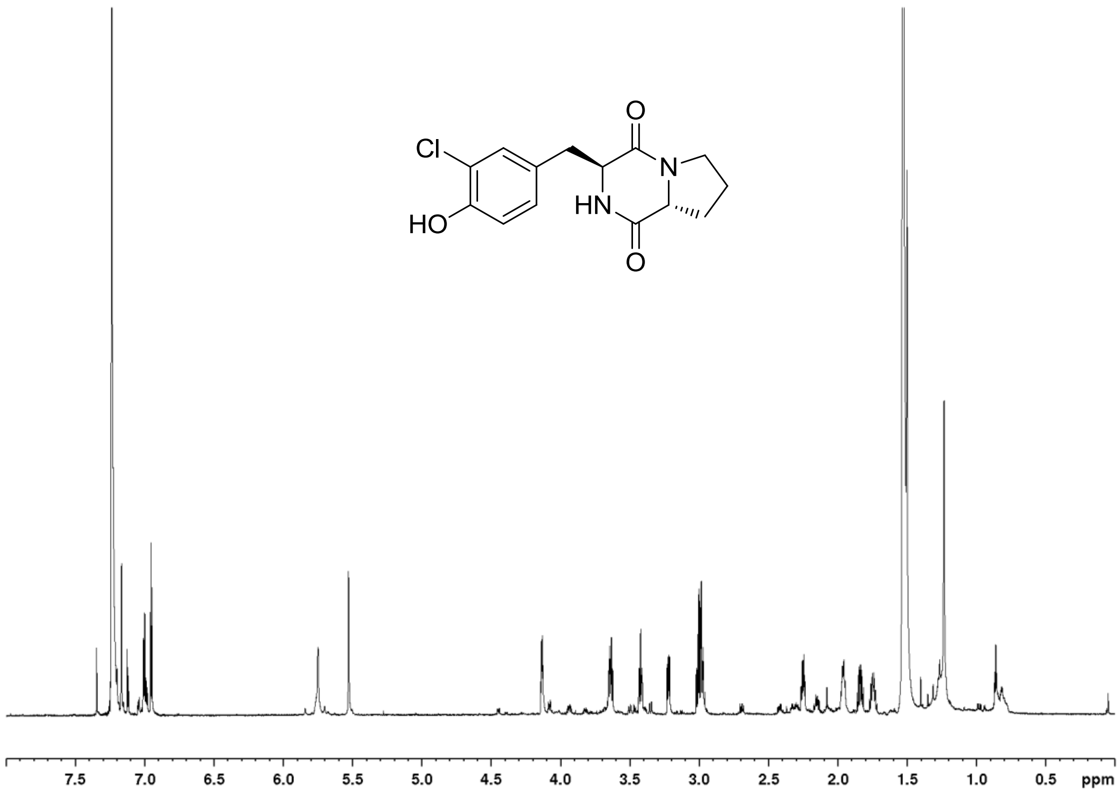

Figure S7. ^{1}H NMR spectrum of *trans*-cyclo(Pro-3-chloro-Tyr) (**16**) in $CDCl_3$.

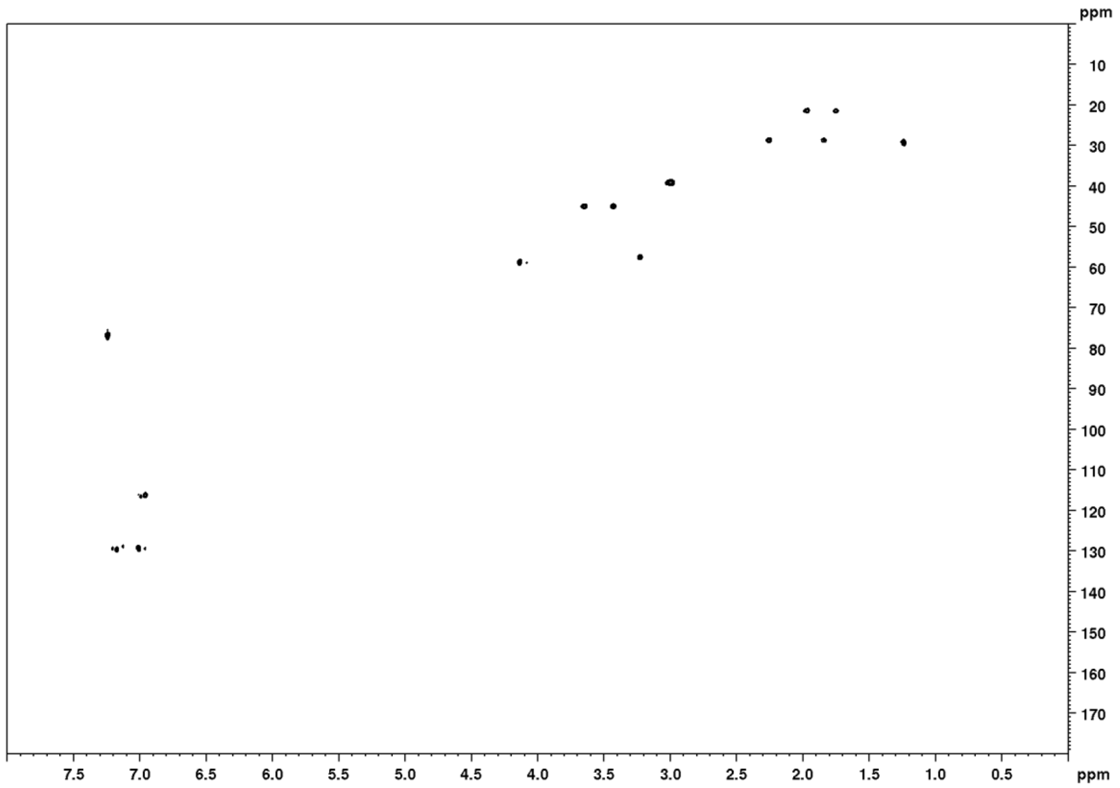

Figure S8. HSQC spectrum of *trans*-cyclo(Pro-3-chloro-Tyr) (**16**) in $CDCl_3$.

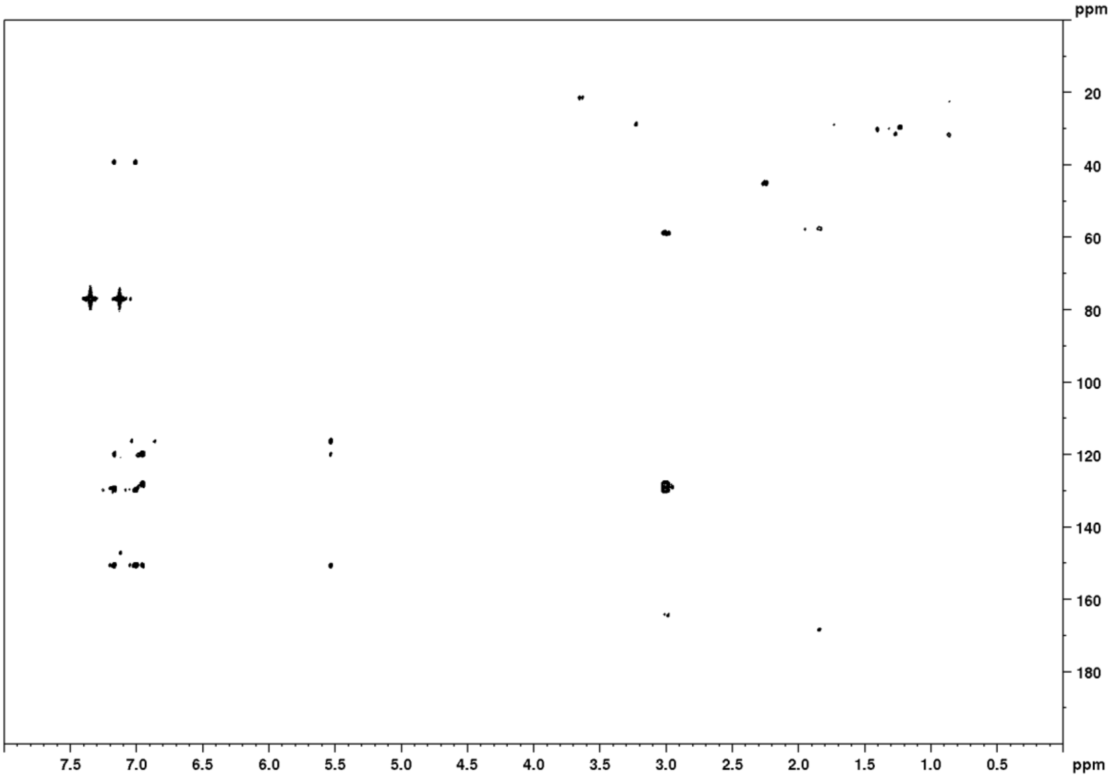

Figure S9. HMBC spectrum of *trans*-cyclo(Pro-3-chloro-Tyr) (**16**) in $CDCl_3$.

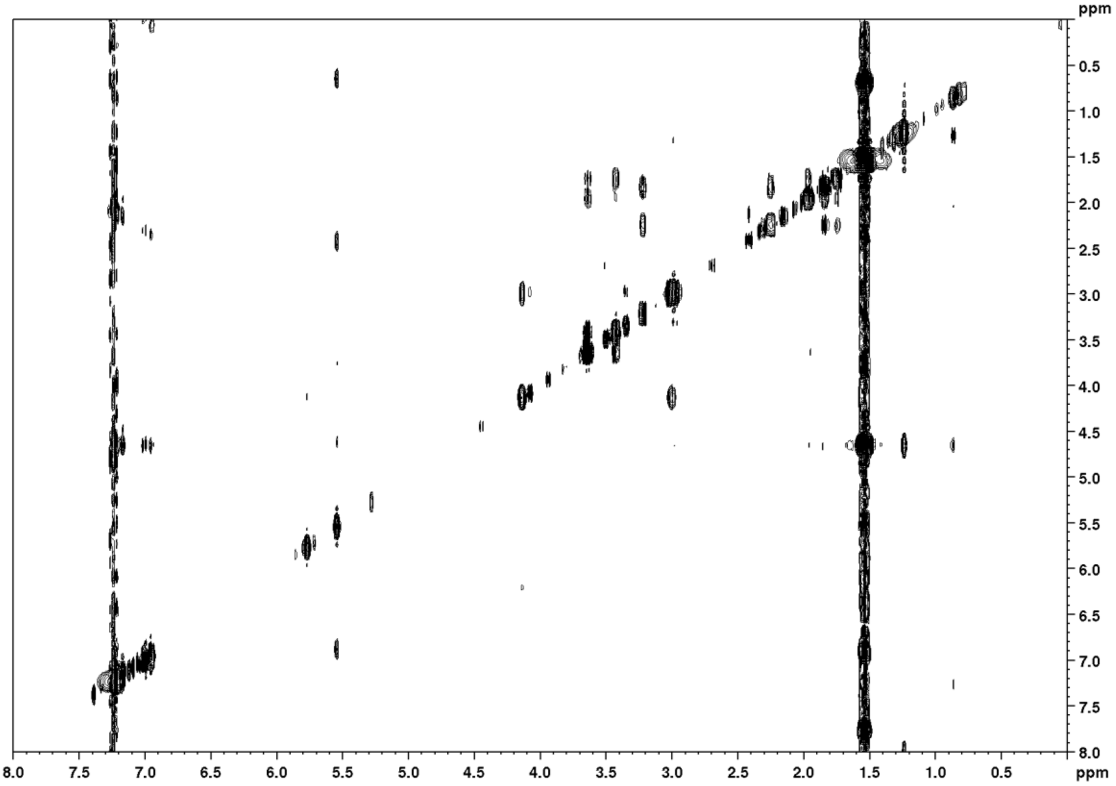

Figure S10. COSY spectrum of *trans*-cyclo(Pro-3-chloro-Tyr) (**16**) in $CDCl_3$.

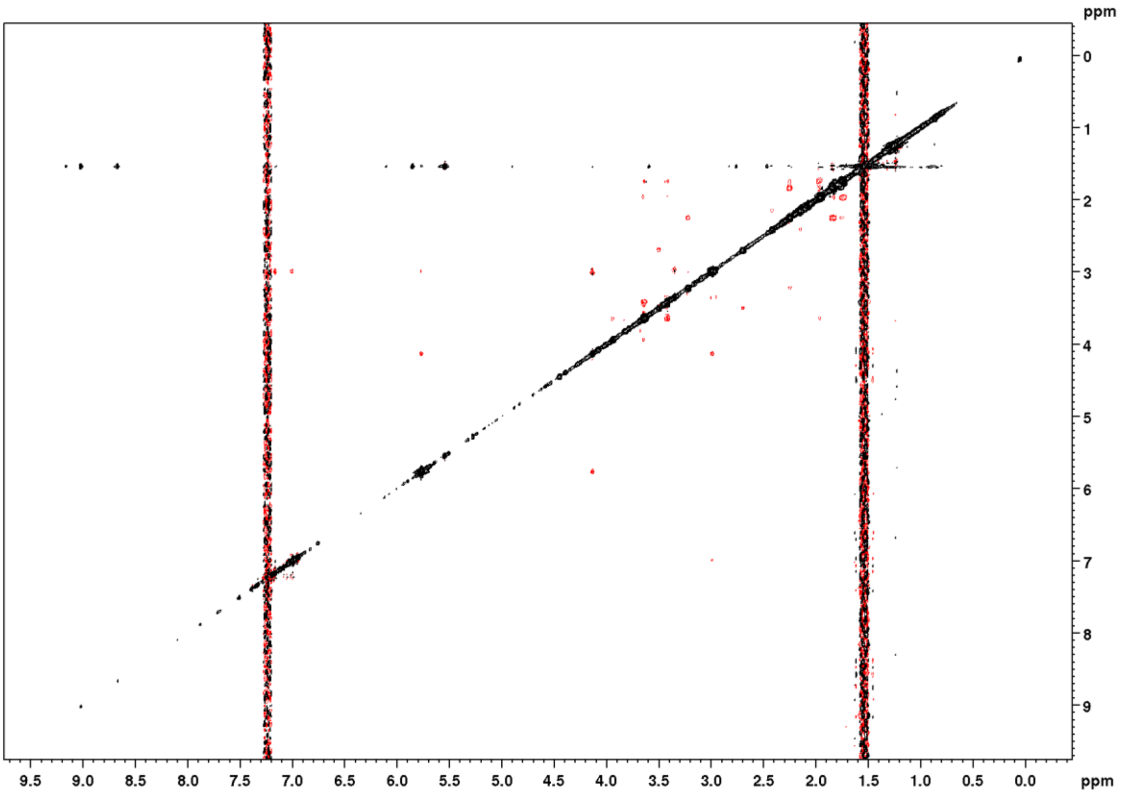

Figure S11. NOESY spectrum of *trans*-cyclo(Pro-3-chloro-Tyr) (**16**) in $CDCl_3$.

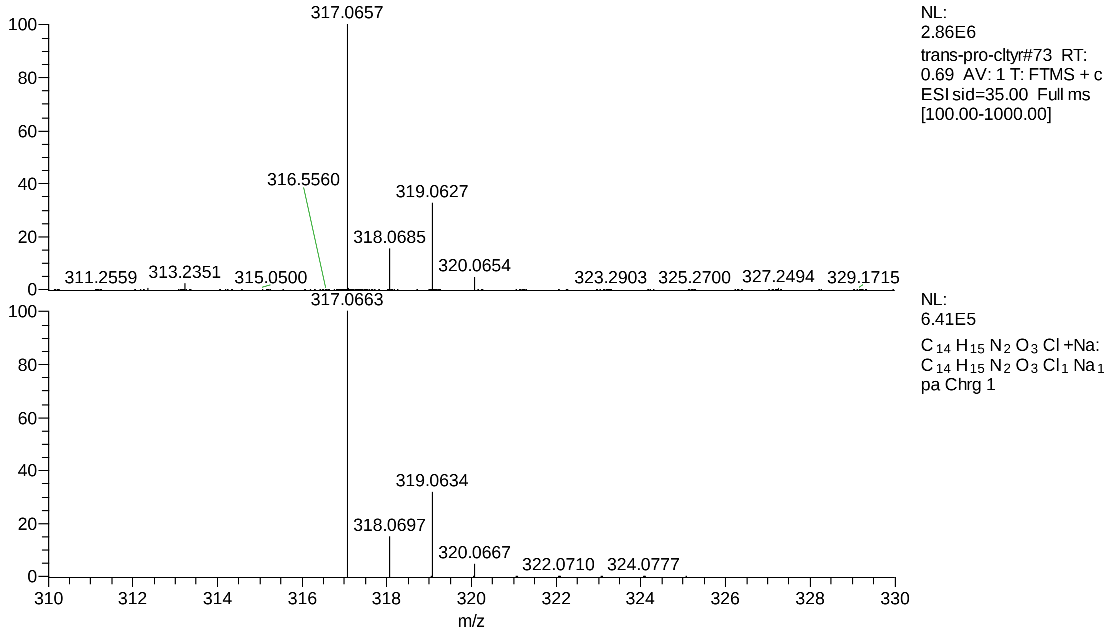

Figure S12. HRESIMS spectrum of *trans*-cyclo(Pro-3-chloro-Tyr) (**16**).

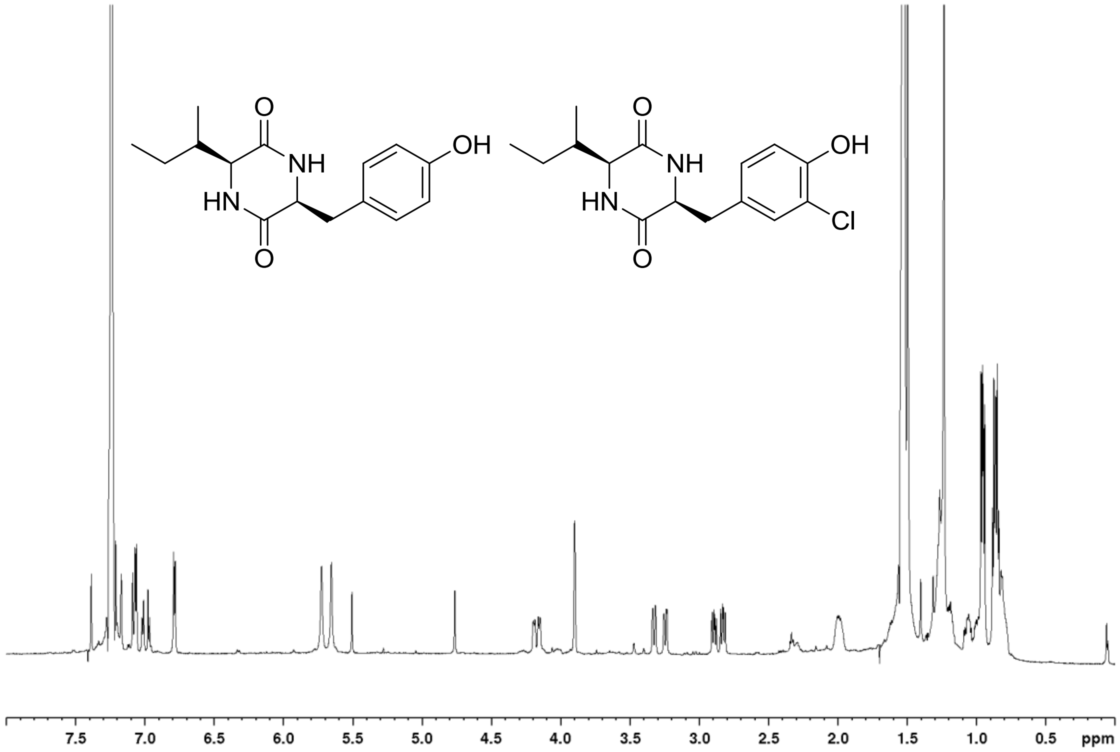

Figure S13. ^{1}H NMR spectrum of the 1:1 mixture of *cis*-cyclo(Tyr-Ile) (**30**) and *cis*-cyclo(3-chloro-Tyr-Ile) (**31**) in $CDCl_3$.

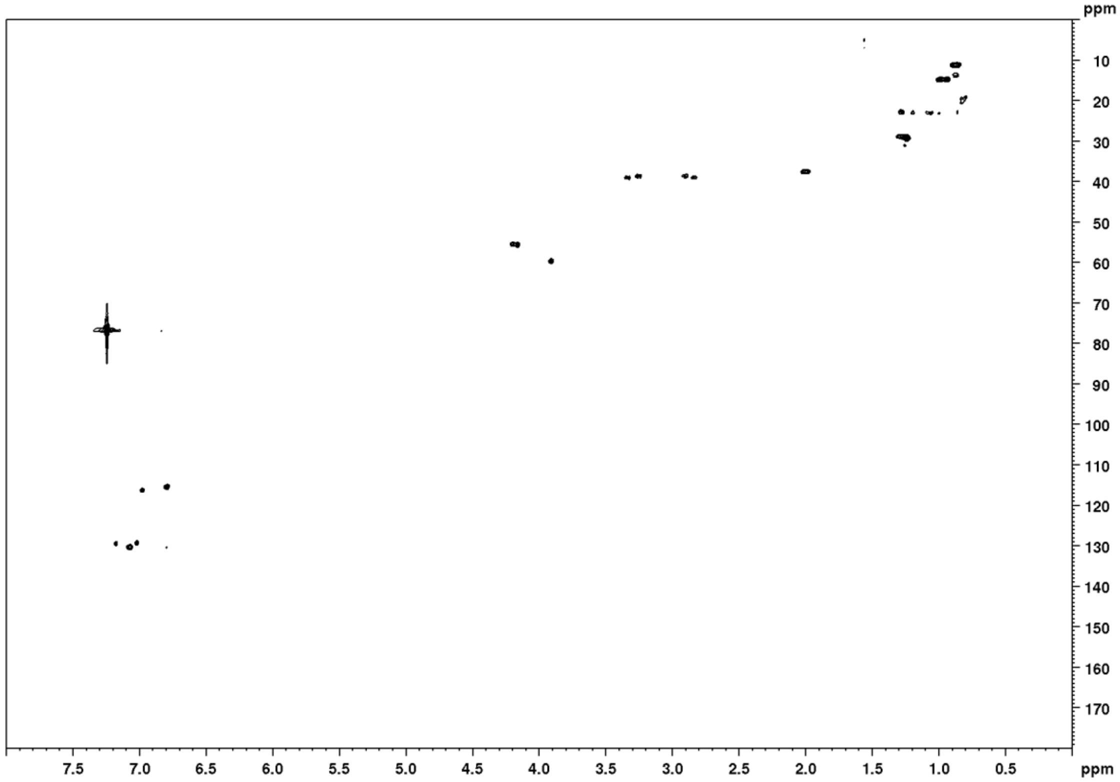

Figure S14. HSQC spectrum of the 1:1 mixture of *cis*-cyclo(Tyr-Ile) (**30**) and *cis*-cyclo(3-chloro-Tyr-Ile) (**31**) in $CDCl_3$.

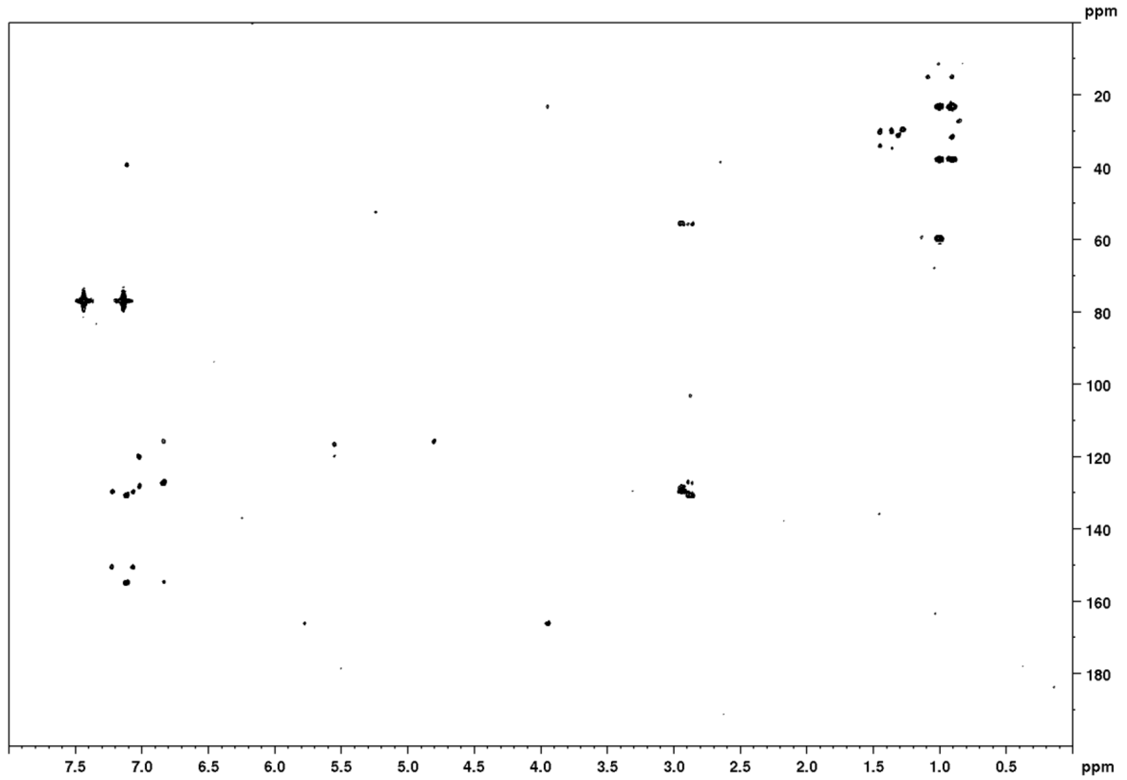

Figure S15. HMBC spectrum of the 1:1 mixture of *cis*-cyclo(Tyr-Ile) (**30**) and *cis*-cyclo(3-chloro-Tyr-Ile) (**31**) in $CDCl_3$.

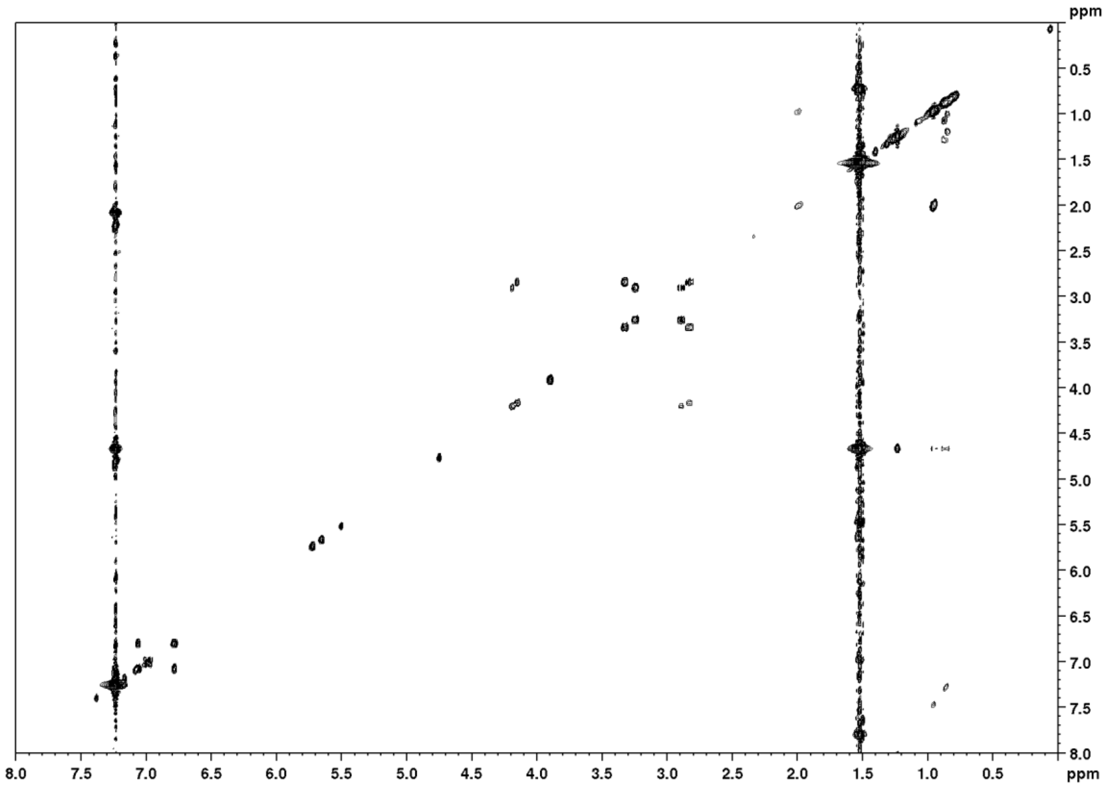

Figure S16. COSY spectrum of the 1:1 mixture of *cis*-cyclo(Tyr-Ile) (**30**) and *cis*-cyclo(3-chloro-Tyr-Ile) (**31**) in $CDCl_3$.

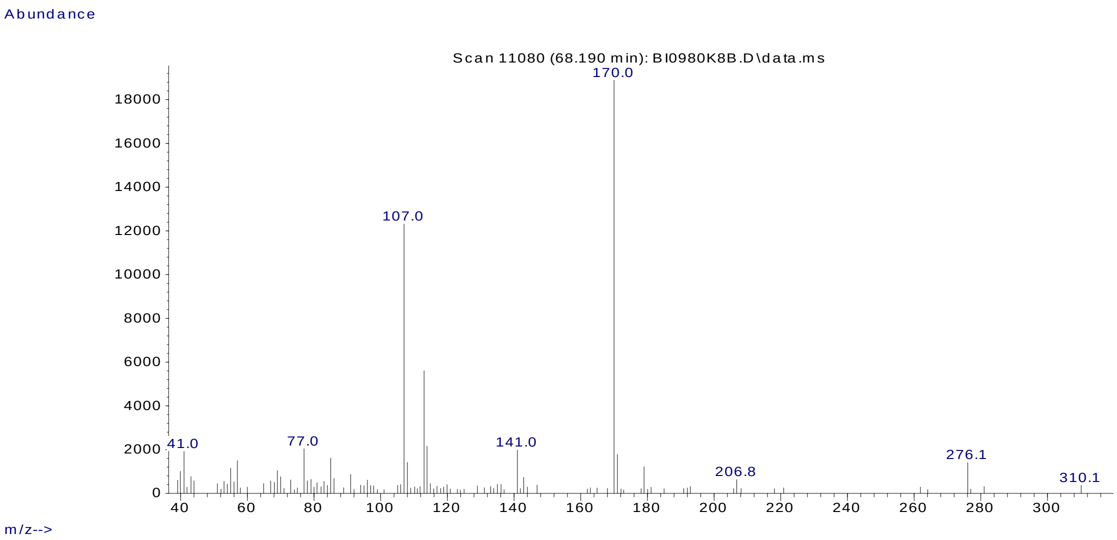

Figure S17. EIMS spectrum of *cis*-cyclo(3-chloro-Tyr-Ile) (**31**).
